# Serial Xenotransplantation in NSG Mice Promotes a Hybrid Epithelial/Mesenchymal Gene Expression Signature and Stemness in Rhabdomyosarcoma Cells

**DOI:** 10.3390/cancers12010196

**Published:** 2020-01-13

**Authors:** Jan Skoda, Jakub Neradil, Iva Staniczkova Zambo, Alena Nunukova, Peter Macsek, Karolina Borankova, Viera Dobrotkova, Pavel Nemec, Jaroslav Sterba, Renata Veselska

**Affiliations:** 1 Department of Experimental Biology, Faculty of Science, Masaryk University, 61137 Brno, Czech Republic; jneradil@sci.muni.cz (J.N.); 416784@mail.muni.cz (A.N.); macsek@mail.muni.cz (P.M.); karolina.borankova@mail.muni.cz (K.B.); 380342@mail.muni.cz (V.D.); 78094@mail.muni.cz (P.N.); 2 International Clinical Research Center, St. Anne’s University Hospital, 65691 Brno, Czech Republic; iva.zambo@fnusa.cz (I.S.Z.);; 3 1st Department of Pathological Anatomy, St. Anne’s University Hospital and Faculty of Medicine, Masaryk University, 65691 Brno, Czech Republic; 4 Department of Pediatric Oncology, University Hospital Brno and Faculty of Medicine, Masaryk University, 62500 Brno, Czech Republic

**Keywords:** rhabdomyosarcoma, cancer stem cells, stemness, stem-like state, serial xenotransplantation, in vivo tumorigenicity assay, epithelial/mesenchymal phenotype

## Abstract

Serial xenotransplantation of sorted cancer cells in immunodeficient mice remains the most complex test of cancer stem cell (CSC) phenotype. However, we have demonstrated in various sarcomas that putative CSC surface markers fail to identify CSCs, thereby impeding the isolation of CSCs for subsequent analyses. Here, we utilized serial xenotransplantation of unsorted rhabdomyosarcoma cells in NOD/SCID gamma (NSG) mice as a proof-of-principle platform to investigate the molecular signature of CSCs. Indeed, serial xenotransplantation steadily enriched for rhabdomyosarcoma stem-like cells characterized by enhanced aldehyde dehydrogenase activity and increased colony and sphere formation capacity in vitro. Although the expression of core pluripotency factors (SOX2, OCT4, NANOG) and common CSC markers (CD133, ABCG2, nestin) was maintained over the passages in mice, gene expression profiling revealed gradual changes in several stemness regulators and genes linked with undifferentiated myogenic precursors, e.g., *SOX4*, *PAX3*, *MIR145*, and *CDH15*. Moreover, we identified the induction of a hybrid epithelial/mesenchymal gene expression signature that was associated with the increase in CSC number. In total, 60 genes related to epithelial or mesenchymal traits were significantly altered upon serial xenotransplantation. In silico survival analysis based on the identified potential stemness-associated genes demonstrated that serial xenotransplantation of unsorted rhabdomyosarcoma cells in NSG mice might be a useful tool for the unbiased enrichment of CSCs and the identification of novel CSC-specific targets. Using this approach, we provide evidence for a recently proposed link between the hybrid epithelial/mesenchymal phenotype and cancer stemness.

## 1. Introduction

Rhabdomyosarcoma is the most common malignant mesenchymal tumor in children, with a peak incidence in patients younger than 5 years old [1]. The embryonal subtype represents ~70% of all rhabdomyosarcoma cases and is associated with a good prognosis. Yet approximately one-third of embryonal rhabdomyosarcoma patients die, mostly from metastatic disease [1,2]. Despite the significant improvement in the survival of rhabdomyosarcoma patients, the prognosis of children with relapsed or metastatic disease has not changed over the last 30 years [2].

As in other cancers, cancer stem cells (CSCs) have been implicated in the recurrence and progression of rhabdomyosarcoma. Although the first studies suggested prominin-1 (CD133) [3,4] or nestin [3] as rhabdomyosarcoma CSC markers, our recent study showed that, regardless of these proteins, only sarcoma cell lines that express high levels of the transcription factor SOX2 form tumors in immunodeficient NOD/SCID gamma (NSG) mice [5]. An in vivo tumorigenicity assay remains a gold-standard functional assay of the CSC phenotype because it enables us to test (directly in an animal model) the ability of cancer cells to self-renew and form a tumor that exhibits the cellular heterogeneity of the primary tumor [6]. Commonly, this assay has been used to determine the tumorigenicity of different cell populations sorted based on prospective CSC surface markers (e.g., CD133, ABCG2, CD24, CD44 [7,8]). Ideally, when a xenograft tumor forms, cells are sorted again and transplanted into secondary recipient mice. This serial xenotransplantation should validate the specificity of a studied marker and verify that the sorted population of cells retains its CSC characteristics [6]. Although it may provide some important evidence, such an approach is to a large extent limited because it assumes sustained levels of marker proteins on the cell surface of CSCs. However, it is becoming evident that the subcellular localization of these putative CSC markers, e.g., CD133 [5,9,10] or CD24 [10,11], is dictated by a complex dynamic process, which presumably reflects cellular needs under different conditions and impairs the use of such proteins as surface markers to distinguish between CSC and non-CSC states [8].

A growing body of evidence shows that enhanced cell plasticity, one of the intrinsic characteristics of CSCs, may provide another explanation for the contradictory results reported regarding the validity of CSC surface markers [7,8,12]. Many of the previously suggested markers have been recently demonstrated as being non-specific, either because both marker-positive and marker-negative cell populations exhibited the same tumorigenic capacities or because marker-positive cells did not form tumors in mice [8]. Thus, recent CSC studies have focused on proteins that are functionally involved in the regulation of stemness and cellular plasticity, such as SOX2, OCT4, or NANOG [8,13]. Indeed, many studies clearly demonstrated a crucial role for these stemness regulators in the induction and maintenance of CSCs in various cancers [8,13,14]. However, because most of these proteins are intracellular, cells must be genetically manipulated to enable their sorting and enrichment according to these markers [13].

Here, we present another approach to study CSCs utilizing an in vivo tumorigenicity assay. It has been previously reported that serial xenotransplantation of cancer cells in immunodeficient mice (passaging in vivo) selects highly tumorigenic cells that generate more aggressive tumors [15,16,17]. Similarly, the acquisition of stemness has been linked with cancer progression and more advanced disease [15]. Thus, we employed serial xenotransplantation of unsorted rhabdomyosarcoma cells as a platform for an unbiased screening of molecular targets that are relevant for the acquisition and maintenance of a CSC phenotype. Our results demonstrate that serial xenotransplantation in NSG mice might be a useful tool for CSC enrichment, allowing subsequent analyses to identify prospective CSC markers and potential therapeutic targets. Using serial xenotransplantation of embryonal rhabdomyosarcoma cells, we revealed an upregulation of several genes associated with stemness and early myogenic precursors. More importantly, this approach allowed us to unveil complex molecular changes that may underlie the induction of stemness in rhabdomyosarcoma, such as a hybrid epithelial/mesenchymal signature, which was recently linked with CSCs in other cancers.

## 2. Results

### 2.1. Tumorigenicity of NSTS-11 Rhabdomyosarcoma Cells Is Maintained during Serial Xenotransplantation in NSG Mice

**Figure 1:**
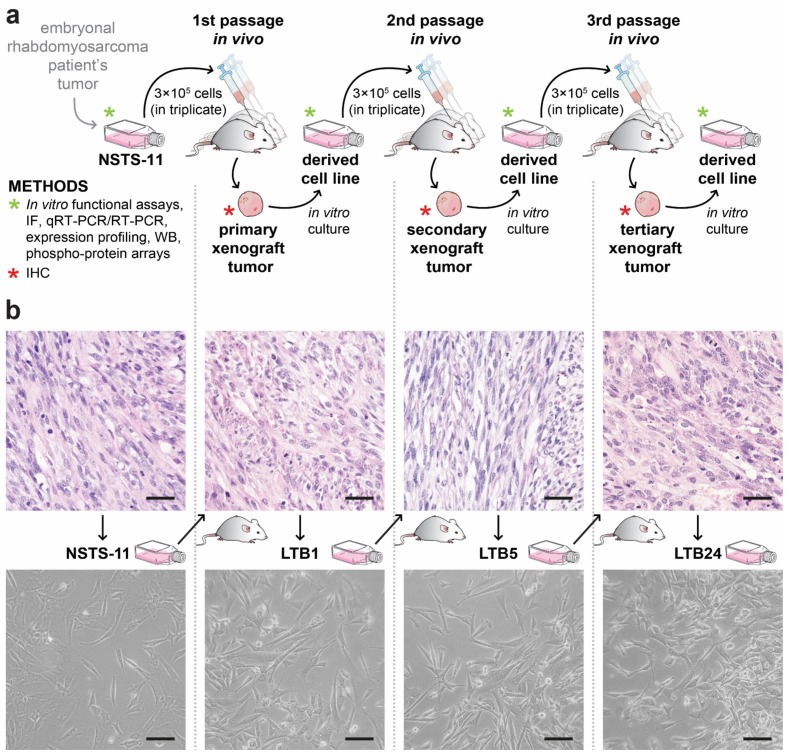
(**a**) Schematic overview of the experimental design. NSTS-11 cells derived from the embryonal rhabdomyosarcoma were injected subcutaneously into three NOD/SCID gamma (NSG) mice (in vivo passage). After the appearance of a tumor, the mouse was sacrificed, and the xenograft tumor was excised and divided into equal parts. One part of the xenograft tumor was used to prepare formalin-fixed paraffin-embedded (FFPE) tissue sections. The second part was processed for primary culture, and the derived cell line was used in a subsequent passage in vivo. This procedure was repeated twice to achieve three in vivo passages. Methods used to analyze tumor tissues or cell lines are indicated. IF, immunofluorescence; qRT-PCR/RT-PCR, real-time/reverse transcription polymerase chain reaction; WB, Western blotting; IHC, immunohistochemistry. (**b**) Representative hematoxylin-eosin images of the tumor tissues (upper panel) and phase contrast microscopy images of the parental NSTS-11 cells and the cells derived from the primary (LTB1), secondary (LTB5), and tertiary (LTB24) xenograft tumors (lower panel). Scale bars, 50 µm (upper panel), 100 µm (lower panel).

The long-term capacity of NSTS-11 cells to form tumors in NSG mice was tested by three subsequent xenotransplantations (passages in vivo; for the experimental design, see Figure 1a). In each passage in vivo, NSTS-11 cells and xenograft tumor-derived cells formed tumors with high efficiency (Table 1). These results confirmed the presence of rhabdomyosarcoma CSCs in the NSTS-11 cell line and demonstrated that these CSCs are maintained during long-term passaging, which included both xenotransplantation in NSG mice and in vitro culture (refer to the experimental design in Figure 1a). The tumorigenicity of 9 cell lines derived from the secondary xenograft tumors was 100%, as revealed in the third, and last, passage in vivo (27 of 27 mice had tumors; Table 1). More importantly, the later in vivo passages resulted in markedly aggressive growth and larger xenografts, as evident from the statistically significant 1.95-fold higher daily increase of the tumor volume in the third passage in vivo compared with the first passage in vivo (2.89 ± 0.74 versus 5.66 ± 0.72 mm^3^/day; Table 1). Hence, tumor growth parameters and tumorigenic efficiency in the third passage in vivo suggested a selection of aggressive tumorigenic cells, i.e., CSCs, during serial xenotransplantation.

To investigate the differences at the cellular and molecular levels that might reflect the selection of CSCs, we decided to analyze three xenograft-derived cell lines and respective tumors that represent one arm of subsequent passages in vivo (Figure 1b). LTB1 (first passage in vivo), LTB5 (second passage in vivo), and LTB24 (third passage in vivo) cell lines and respective tumors were included for further analyses; NSTS-11 cells and the primary tumor served as a parental control.

### 2.2. The Colony Formation and Sphere Formation Capacity of NSTS-11 Cells Is Enhanced after In Vivo Passages

**Figure 2:**
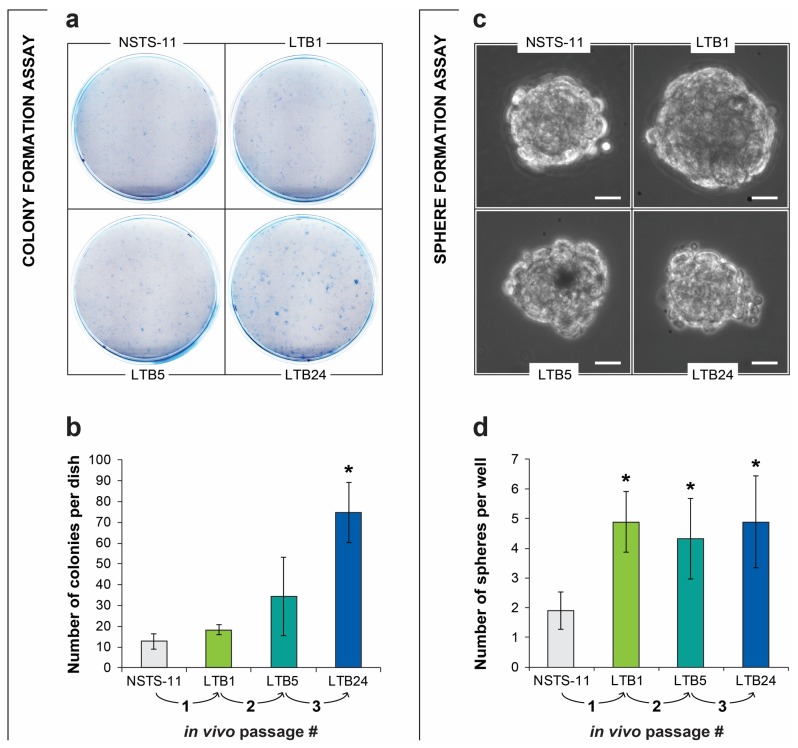
In vitro functional assays revealed an enrichment of cancer stem cells (CSCs) over in vivo passages. (**a**) Representative images of the colony formation assay. A total of 1000 cells were plated per culture dish and cultured for 8 days. (**b**) Quantification of the colonies (>50 cells per colony) formed by the parental NSTS-11 cells and the cells derived from the primary (LTB1), secondary (LTB5), and tertiary (LTB24) xenograft tumors. Data are presented as the mean ± SD of three independent experiments. (**c**) Representative images of rhabdospheres formed by the respective cell lines. Scale bars, 25 µm. (**d**) More than a two-fold increase in sphere formation capacity over in vivo passages. The number of spheres is presented as the mean ± SD of three independent experiments. * Significantly higher compared with the parental NSTS-11 cells (*p* < 0.05).

To evaluate how the serial xenotransplantation of NSTS-11 cells affects their CSC characteristics in vitro, we first assayed the capacity of LTB1, LTB5, and LTB24 cells to form colonies and spheres compared with that of parental NSTS-11 cells (Figure 2). The results of these in vitro functional assays of CSCs revealed an increase in both colony and sphere formation capacity over in vivo passages, which indicates an enrichment of CSCs. Tertiary xenograft tumor-derived LTB24 cells formed significantly more colonies (Figure 2a,b) and rhabdospheres (Figure 2c,d) in vitro than parental NSTS-11 cells. The upward trend in the number of colonies and increased sphere formation capacity supported our in vivo observations and implied that CSCs are enriched during serial xenotransplantation. Importantly, together with the in vivo tumorigenicity assay, the colony formation assay and sphere formation assay confirmed that CSCs are maintained in low passages of xenograft-derived cell lines cultured in vitro.

### 2.3. Serial Xenotransplantation in NSG Mice Increases Aldehyde Dehydrogenase Activity In Vitro

**Figure 3:**
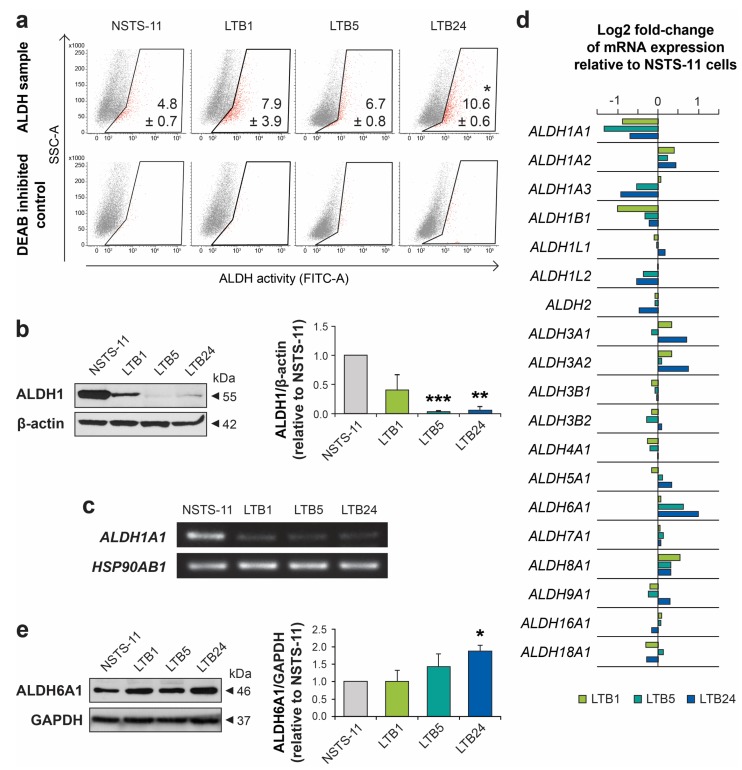
Aldehyde dehydrogenase activity and expression analysis. (**a**) Aldefluor™ assay showed an increase in aldehyde dehydrogenase (ALDH) activity over in vivo passages. Representative dot plots are shown. The percentage of ALDH-positive cells is presented as the mean ± SD of three independent experiments. * Statistically significant difference from parental NSTS-11 cells (*p* < 0.05). (**b**) Western blot analysis of ALDH1 expression. β-actin served as a loading control. Representative image (left) and mean relative optical density values ± SD (right) of three independent experiments. ** *p* < 0.01, *** *p* < 0.001. (**c**) Expression of the *ALDH1A1* gene as detected by RT-PCR. *HSP90AB1* served as a control. (**d**) Microarray gene expression analysis of ALDH gene variants. (**e**) Western blot analysis confirmed upregulation of ALDH6A1 protein over in vivo passages. Glyceraldehyde-3-phosphate dehydrogenase (GAPDH) served as a loading control. Representative image (**left**) and mean relative optical density values ± SD (**right**) of three independent experiments. * *p* < 0.05.

High aldehyde dehydrogenase (ALDH) activity has been attributed to CSCs in many cancers, including rhabdomyosarcoma [18]. Therefore, we employed an Aldefluor™ assay to functionally characterize changes in ALDHs during in vivo passaging. In agreement with the previous in vitro assays of CSCs, the Aldefluor™ assay demonstrated a significant increase in ALDH activity in LTB24 cells and revealed a trend of gradually increasing ALDH activity over in vivo passages (Figure 3a). Surprisingly, analysis of the ALDH1 enzyme showed its downregulated expression in later xenograft-derived cell lines (Figure 3b and Figure S1). Similarly, RT-PCR demonstrated downregulated expression of the *ALDH1A1* gene (Figure 3c), which encodes one of the prominent ALDH1 isoforms commonly associated with CSCs [19]. We therefore analyzed expression profiling data and compared the expression levels of each of the 19 ALDH gene variants (Figure 3d). Expression profiling confirmed the downregulation of several members of the ALDH1 family, mainly *ALDH1A1* and *ALDH1A3*. However, the analysis revealed upregulated expression of some ALDH gene variants, especially *ALDH6A1*, which may account for the Aldefluor™ assay results, as discussed later. The significant upregulation of ALDH6A1 variant was further confirmed also at protein levels (Figure 3e and Figure S2).

### 2.4. Expression Levels of Core Pluripotency Factors and Common CSC Markers Are Preserved Over the Passages In Vivo

Our previous study showed the key role of the pluripotency factor SOX2 in sarcoma tumorigenesis [5]; thus, we aimed to investigate whether the enrichment of CSCs during serial xenotransplantation was accompanied by changes in expression of the core pluripotency factors (SOX2, OCT4, and NANOG) or commonly used CSC markers (nestin, CD133, and ABCG2). Immunohistochemistry (IHC) of tumor tissues did not show any evidence of gradual selection for any of the evaluated proteins (Table 2, Figures S3 and S4). The expression levels of these proteins were maintained in the tumor tissues over the passages in vivo, except for minor changes in SOX2 and CD133 expression.

**Figure 4:**
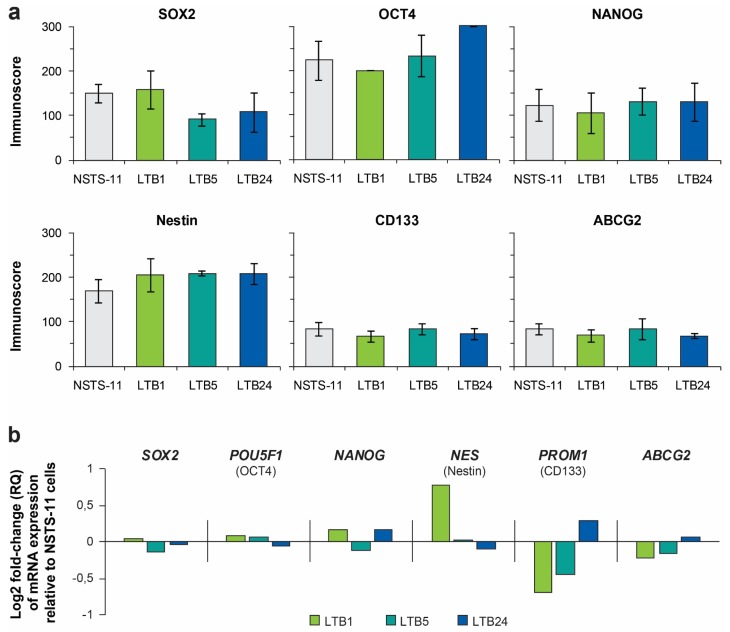
(**a**) Immunofluorescence analysis of core pluripotency factors (upper panel) and commonly used CSC markers (lower panel). Immunoscores were determined by multiplying the percentage of positive cells by the respective immunoreactivity. Data are presented as the mean ± SD of at least three independent experiments. (**b**) Expression profiling data of genes encoding the investigated proteins. Where necessary, the protein name is noted under the official gene symbol.

In agreement with the IHC results, immunofluorescence analysis did not show any significant difference in expression of SOX2, OCT4, NANOG, nestin, CD133, or ABCG2 among the derived cell lines (Figure 4a, Figures S5 and S6). In addition, no clear trend in the expression of these proteins at the mRNA level was identified using gene expression profiling (Figure 4b) and further validated using RT-PCR (Figure S7). Together, these results suggested that the expression of the six investigated proteins did not account for the enhanced stemness observed during serial xenotransplantation in NSG mice and that other genes or mechanisms were involved. However, a sustained expression of most of these proteins confirms their important roles in rhabdomyosarcoma tumorigenesis.

### 2.5. Serial Xenotransplantation of Rhabdomyosarcoma Cells Promotes an Expression Profile Associated with Muscle Progenitor Cells and a Hybrid Epithelial/Mesenchymal Phenotype

**Figure 5:**
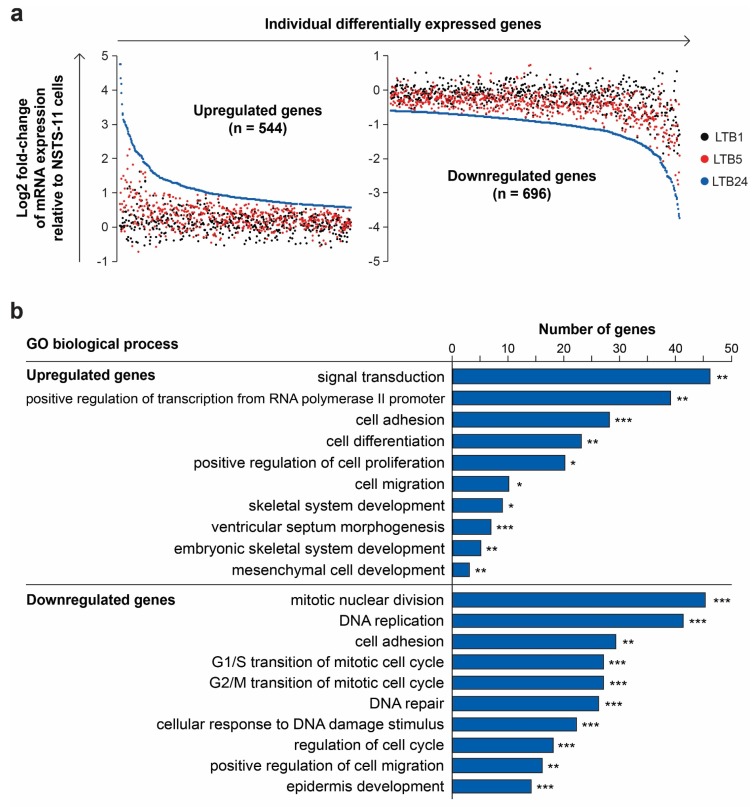
Analysis of the differentially expressed genes. (**a**) Expression levels of differentially expressed genes identified by their upward (upregulated genes; left plot) or downward (downregulated genes; right plot) trend of expression over passages in vivo; (**b**) Gene ontology (GO) analysis of biological processes. The DAVID annotation tool with the GOTERM_BP_DIRECT database was used. Modified Fisher’s exact test, * *p* < 0.05, ** *p* < 0.01, and *** *p* < 0.001.

To identify genes that may account for the observed enrichment of cells with CSC characteristics during serial passaging in vivo, we performed gene expression profiling of NSTS-11, LTB1, LTB5, and LTB24 cell lines. First, we defined two groups of differentially expressed genes (refer to Section 4 for the detailed procedure; Table S1). The group of upregulated genes (*n* = 544) included only genes that were significantly upregulated after the third in vivo passage and exhibited an upward trend in expression during serial xenotransplantation (Figure 5a). Conversely, the group of downregulated genes (*n* = 696) comprised only significantly downregulated genes that followed a downward trend of expression (Figure 5a). Analysis of these two groups of genes allowed us to determine the expression profile that was gradually selected over the passages in vivo and might be associated with CSCs in embryonal rhabdomyosarcoma.

Gene Ontology term enrichment analysis (Table S1) showed that upregulated genes were involved in biological processes comprising signal transduction, cell adhesion, and migration, positive regulation of transcription and cell proliferation, and several developmental processes (Figure 5b). Downregulated genes were enriched in processes such as cell cycle and DNA repair regulation, cell adhesion and migration, and epidermis development (Figure 5b). Many of the downregulated genes, which were included in the epidermis development term, encoded keratins (Table S1), therefore we hypothesized that serial xenotransplantation induced epithelial-mesenchymal transition (EMT) program in NSTS-11 cells. A significant enrichment of “cell migration” and “cell adhesion” ontology terms among both downregulated and upregulated genes (Figure 5b) further encouraged our hypothesis.

**Figure 6:**
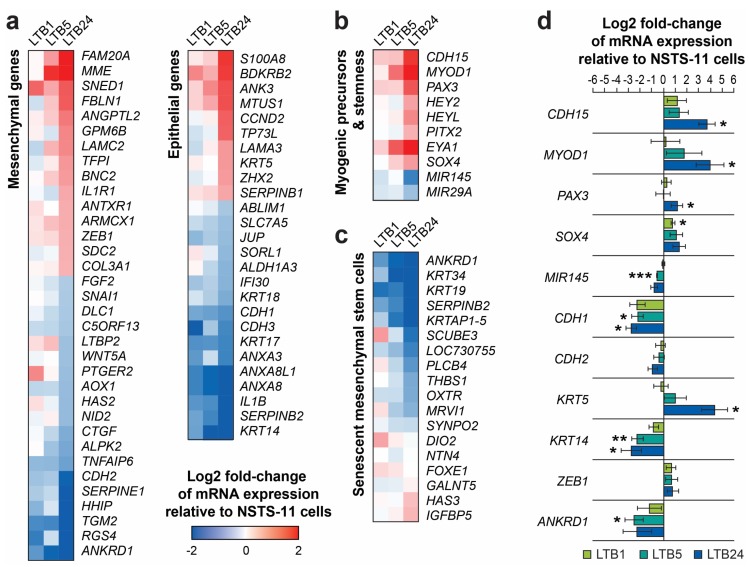
(**a**) Expression levels of differentially expressed genes categorized based on their association with a mesenchymal or an epithelial phenotype. (**b**) Expression profile signature suggesting enhanced stemness and impaired myogenic differentiation. (**c**) Expression profile of the genes that are associated with senescence of mesenchymal stem cells [25]. (**d**) qRT-PCR validation of the gene expression profiling data. The expression of all 11 genes tightly reflected the expression profile detected by microarray analysis (see **a**,**b**). Data are presented as the mean ± SD of three biological replicates. * *p* < 0.05, ** *p* < 0.01, and *** *p* < 0.001 relative to parental NSTS-11 cells.

To test this hypothesis, we created a list of EMT-related genes by combining two published gene sets that were identified based on a review of the literature [20] and a meta-analysis of gene expression studies [21] (Table S1). The list contains a set of genes associated with mesenchymal traits (mesenchymal genes) and a set of genes commonly associated with epithelial traits (epithelial genes). This list allowed us to categorize the differentially expressed genes accordingly (Figure 6a). Of the mesenchymal genes (*n* = 126), 15 genes, including *ZEB1*, *MME*, *LAMC2*, or *COL3A1*, were found to be upregulated, whereas 19 genes, including *CDH2* (N-cadherin), *SNAI1*, *FGF2*, *AOX1*, or *ANKRD1*, were downregulated (Figure 6a). Of the epithelial genes (*n* = 137), 16 genes, including *CDH1* (E-cadherin), *CDH3* (P-cadherin), *KRT14*, *KRT17*, or *KRT18*, were downregulated over the passages in vivo, whereas 10 genes, such as *KRT5*, *LAMA3*, or *ANK3,* were upregulated (Figure 6a). In total, the expression of 60 genes (22.8%) out of 263 EMT-related genes was significantly and gradually shifted during serial xenotransplantation. However, the functional distribution of these genes equally indicated a transition to both mesenchymal (31 genes) and epithelial (29 genes) phenotypes (Figure 6a). These results suggest that the acquisition of aggressive stem-like traits was associated with the selection of cells that exhibit a hybrid epithelial/mesenchymal phenotype (mixed epithelial and mesenchymal gene expression signature).

According to the gene ontology analysis, several developmental processes were enriched for upregulated genes that encode transcription factors, such as *SOX4*, *HEYL*, *HEY2*, or *PITX2*, and genes involved in TGFβ and BMP signaling, i.e., *TGFBR3* and *NOG* (Table S1). Based on a review of the literature, we focused on genes related to stemness and muscle progenitor cells (satellite cells). Indeed, we identified several upregulated myogenic transcription factors that are associated with quiescent/undifferentiated muscle precursors (Figure 6b). *PAX3* [22], *HEYL*, *HEY2*, and *PITX2* [23] were identified among the most prominent differentially expressed genes. Furthermore, markers of satellite cells, CDH15 (M-cadherin), and of the myogenic cell lineage, *MYOD1*, were also upregulated. Importantly, the expression pattern of several stemness regulators provided further evidence for the progressive dedifferentiation and/or enhanced stemness of NSTS-11 cells over the passages in vivo. In addition to the upregulation of *SOX4* and *EYA1*, we identified the downregulation of two microRNA genes, *MIR29A* and a well-known stemness inhibitor *MIR145* [8,24] (Figure 6b). Further analysis of 18 genes that correlate with mesenchymal stem cell senescence [25] revealed a remarkably significant downregulation of 10 genes (*ANKRD1*, *KRT34*, *KRT19*, *SERPINB2*, *KRTAP1-5*, *LOC730755*, *PLCB4*, *THBS1*, *OXTR*, *MRVI1*), while none of the analyzed genes was significantly upregulated during serial xenotransplantation (Figure 6c). Overall, these results clearly suggest that serial xenotransplantation in NSG mice selected for rhabdomyosarcoma cells (presumably CSCs), which acquire an expression signature of primitive undifferentiated cells that resemble non-senescent myogenic precursors/mesenchymal stem cells.

To validate the gene expression profiling results, we performed qRT-PCR of 11 genes associated with EMT, stemness, or muscle progenitor cells, as described above (Figure 6d). Indeed, qRT-PCR using three biological replicates (different cell passages in vitro) confirmed the trends in expression of all the examined genes previously identified by gene expression profiling. This result demonstrates the validity of the gene expression profiling data in our study and underpins the stability of gene expression signatures during in vitro culture.

### 2.6. Serial Passaging In Vivo Enhanced the Phosphorylation of the Wnt Coreceptor RYK

As gene expression profiling revealed a gradual upregulation of several genes involved in cell signaling and signal transduction (e.g., *PDGFRB*, *ROR2*, or *FGF18*; Figure 5b and Table S1), we next investigated whether this led to the activation of receptor tyrosine kinases (RTKs) and their downstream signaling pathways. To assess the levels of phosphorylation of various RTKs, we performed a phospho-RTK antibody array using cells cultured for 24 h in either serum-free or complete media. Unexpectedly, regardless of the culture conditions, the phosphorylation of most of the RTKs did not differ among NSTS-11 and xenograft tumor-derived cell lines (Figure S8). However, one of the most abundantly phosphorylated RTKs in our study, receptor like tyrosine kinase (RYK), showed a gradual increase (>2.5-fold) in phosphorylation during serial xenotransplantation of NSTS-11 cells in NSG mice (Figure S8). This increase in the level of phosphorylated RYK was not caused by upregulated gene expression, as *RYK* mRNA levels remained unchanged (Table S1).

RYK is an atypical RTK with impaired kinase activity [26,27] but functions as a coreceptor in Wnt signaling, which is implicated in self-renewal and stemness [28]. Notably, RYK has also been demonstrated to activate mitogen-activated protein kinases (MAPKs), i.e., ERK1/2 [26]. In line with these findings, we detected an apparent upregulation of phosphorylated ERK1 using phospho-MAPK antibody arrays (Figure S9). Compared with parental NSTS-11 cells in both serum- and serum-free conditions, the levels of phosphorylated ERK1 were upregulated in LTB5 and LTB24 cells derived from secondary and tertiary xenografts, respectively (Figure S9). These results suggest positive regulation of the MAPK/ERK1 cascade via enhanced Wnt/RYK signaling. Gene expression profiling of the xenograft-derived cell lines further revealed upregulated mRNA levels of several Wnt receptors and coreceptors, including *FZD3*, *FZD4*, *FZD8*, and *ROR2*, and identified a significant increase in the expression of the Wnt downstream target gene *WISP2/CCN5* (Table S1). High expression levels of *WISP2* were reported in undifferentiated mesenchymal stem cells, and Wisp2 was shown to increase mesenchymal precursor cell proliferation in vitro [29,30]. Wisp2 also induces a dedifferentiated state in adipose cells favoring the myofibroblast phenotype [30]. Together, the antibody arrays and gene expression profiling identified a marked modulation of Wnt signaling during serial xenotransplantation and suggested RYK as a prospective target in rhabdomyosarcoma CSCs.

### 2.7. Expression of Several Identified Prospective CSC-Specific Target Genes Predicts Survival in Soft-Tissue Sarcomas

Using serial xenotransplantation in conjunction with gene expression profiling and antibody arrays, we identified several prospective targets that might be associated with the progression of rhabdomyosarcoma and the acquisition of a CSC phenotype. Hence, we applied in silico analysis of publicly available gene expression data to test the relevance of our model.

**Figure 7:**
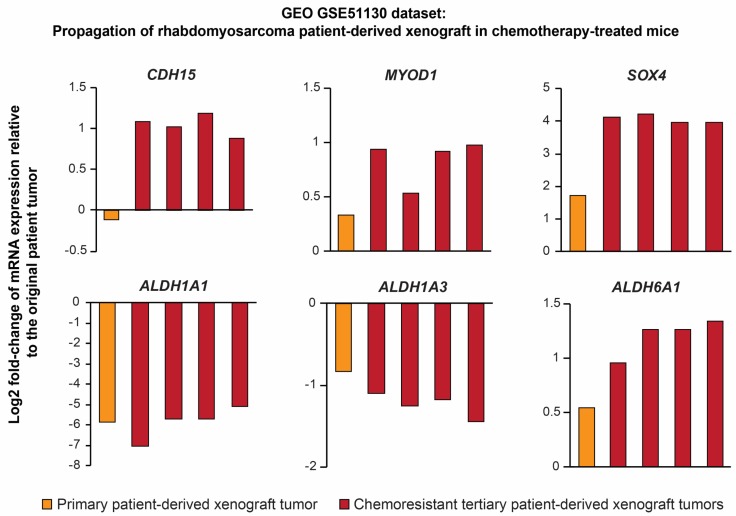
Gene expression profiles of aggressive chemotherapy-resistant rhabdomyosarcoma patient-derived xenografts (Gene Expression Omnibus (GEO) GSE51130 dataset) resemble the stemness-associated gene expression signature identified using our approach. Note the marked downregulation of *ALDH1A1* and upregulation of *ALDH6A1* in refractory chemoresistant xenografts (red bars; expression for individual biological replicates) compared with the original patient tumor.

First, we performed an analysis of Gene Expression Omnibus GSE51130 dataset, which comprises expression profiles of primary rhabdomyosarcoma and xenograft tumors obtained during serial propagation of patient-derived xenograft (PDX) in mice treated with standard chemotherapy (Figure 7). Supporting our model, gene expression profiles of the refractory chemoresistant rhabdomyosarcoma PDXs strikingly corresponds with the stemness-associated gene expression signature identified in our study, including significant upregulation of *ALDH6A1*, *SOX4*, *CDH15*, and *MYOD1* expression as well as downregulation of *ALDH1A1* and *ALDH1A3* (Figure 7).

To the best of our knowledge, no rhabdomyosarcoma-specific gene expression dataset paired with clinical follow-up data was available at the time of the analysis. Thus, we utilized The Cancer Genome Atlas Sarcoma (TCGA-SARC) dataset, which includes various soft-tissue sarcomas, to evaluate the clinical relevance and prognostic significance of the prospective rhabdomyosarcoma CSC-associated genes identified in our model.

In line with our previous results [5], survival analysis revealed that among *SOX2*, *POU5F1* (encoding OCT4), *NANOG*, *PROM1* (encoding CD133), *NES* (encoding nestin), and *ABCG2* genes, only *SOX2* expression was significantly (*p* < 0.05) associated with the survival of sarcoma patients and predicted poor prognosis (Figure S10). In the present study, we also observed a slight increase in the number of SOX2-positive cells in the xenograft tissues (Table 2; Figure S3), which further supports that SOX2 plays a major role in sarcoma tumorigenesis and the maintenance of CSCs [8].

**Figure 8:**
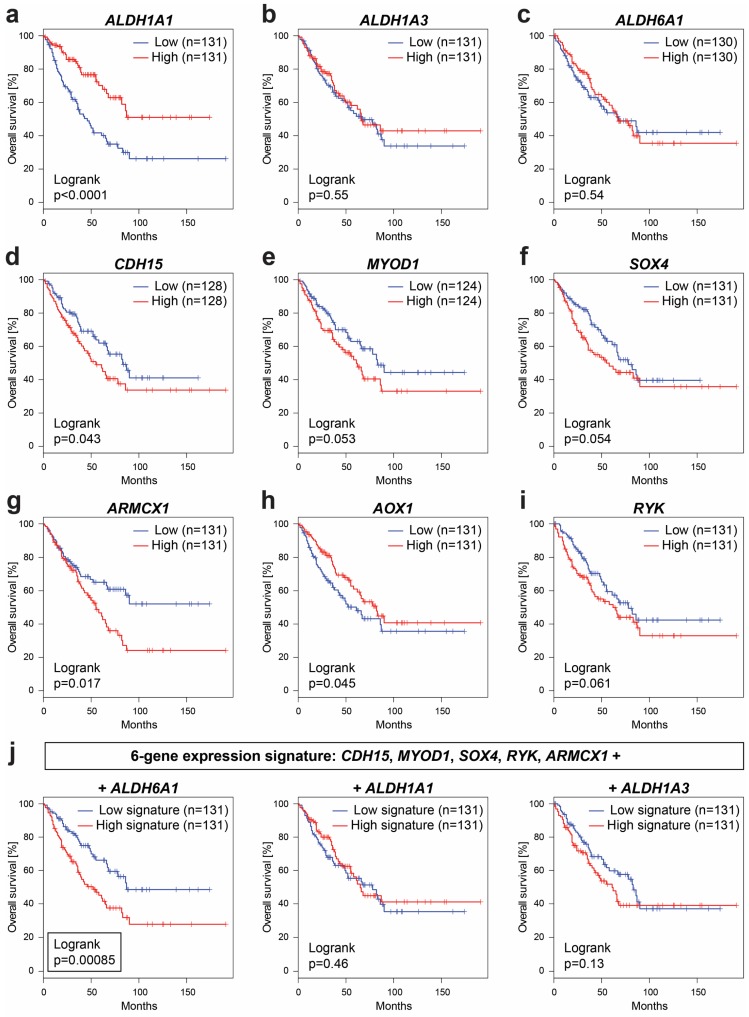
Overall survival in soft-tissue sarcomas (The Cancer Genome Atlas Sarcoma (TCGA-SARC) dataset) based on the gene expression levels of (**a**) *ALDH1A1*, (**b**) *ALDH1A3*, (**c**) *ALDH6A1*, (**d**) *CDH15*, (**e**) *MYOD1*, (**f**) *SOX4*, (**g**) *ARMCX1*, (**h**) *AOX1*, and (**i**) *RYK*. (**j**) Significant prognostic value of 6-gene expression signature comprising *ALDH6A1* (**left**) but not *ALDH1A1* (**middle**) and *ALDH1A3* gene (**right**). The analysis was performed using the GEPIA 2 online tool [32].

More importantly, TCGA-SARC analysis revealed a marked prognostic potential of several identified target genes, which were gradually modulated during serial xenotransplantation and associated with the increased stemness of rhabdomyosarcoma cells. In agreement with the downregulation of *ALDH1A1* detected in the xenograft-derived cell lines (Figure 3c,d), low levels of *ALDH1A1* expression are strongly correlated (*p* < 0.0001) with short overall survival among patients with soft-tissue sarcomas (Figure 8a). These striking results suggest that ALDH1A1 may play different roles in the tumorigenesis and stemness of sarcomas than were commonly reported in carcinomas [19]. In contrast, the expression of *ALDH1A3* (Figure 8b) or *ALDH6A1* (Figure 8c) alone cannot predict survival in soft-tissue sarcoma patients, but the differential expression of these genes might be specific for rhabdomyosarcoma as indicated by PDX expression profiling data (Figure 7) and by a previous study that reported *ALDH6A1* to be associated with worse survival in rhabdomyosarcoma patients [31].

Of the highly expressed myogenic precursor and stemness-related genes, *CDH15* (Figure 8d) was identified as a significant (*p* < 0.05) predictor of poor prognosis in soft-tissue sarcomas along with *MYOD1* (Figure 8e) and SOX4 (Figure 8f), which, however, did not reach the level of statistical significance (*p* = 0.053 and *p* = 0.054, respectively). Among the identified genes linked with mesenchymal traits, expression of the *ARMCX1* gene, which encodes mitochondria-localized armadillo repeat-containing X-linked protein 1, was revealed as a very strong predictor of poor outcome in soft-tissue sarcomas (Figure 8g). Conversely, the expression of another mesenchymal gene, *AOX1*, which was downregulated during serial xenotransplantation (see Figure 6a), significantly correlates with a better prognosis in sarcoma patients (Figure 8h). These results provide exemplary evidence that sarcoma progression may not be driven by an enhanced mesenchymal gene expression but rather by a hybrid epithelial/mesenchymal expression signature that reflects the activation of both epithelial and mesenchymal programs.

In agreement with the results of phospho-RTK arrays, the upregulated expression of *RYK* tends to correspond with worse prognosis (Figure 8i). However, the prognostic power of *RYK* expression is fully pronounced when analyzed in combination with the expression of other prospective CSC-related genes that nearly reached statistical significance in the survival analysis. In fact, the expression signature of six genes, including *CDH15*, *MYOD1*, *SOX4*, *ARMCX1*, *RYK*, and *ALDH6A1*, was identified as a strong predictor of poor survival (*p* < 0.001) that markedly overcomes statistical significance of the individual genes (Figure 8j). On the contrary, gene signatures comprising *ALDH1A1* or *ALDH1A3* instead of *ALDH6A1* failed to predict survival (Figure 8j). These results suggest that it is the *ALDH6A1* gene (not *ALDH1A1* or *ALDH1A3*) that is more likely upregulated together with *CDH15*, *MYOD1*, *SOX4*, *ARMCX1*, and *RYK* in aggressive soft-tissue sarcomas.

It is important to note, that TCGA-SARC soft-tissue sarcoma dataset does not contain rhabdomyosarcoma samples, which poses some limitations for the interpretation of the survival data in regard to this tumor type. However, the marked level of agreement between our experimental results and the survival data of various soft-tissue sarcomas suggests that our approach for CSC enrichment could be a useful tool for the unbiased identification of molecular targets that might be associated with enhanced stemness and poor prognosis in rare cancers, such as embryonal rhabdomyosarcoma.

## 3. Discussion

Bottom-up approaches that would enrich for CSCs based on their functional characteristics and might allow the objective screening of CSC-specific markers and therapeutic targets are currently of broad interest to many cancer researchers. Here, we report a proof-of-principle platform to study rhabdomyosarcoma CSCs that successfully combines serial xenotransplantation in vivo with short-term culture of xenograft-derived cells in vitro.

A fundamental observation made while testing our approach is that rhabdomyosarcoma CSCs are maintained during short-term in vitro culture of xenograft-derived cell lines, which was confirmed by the high tumorigenicity of primary and secondary xenograft-derived cells. In fact, as serial xenotransplantation selected for CSCs, their numbers in xenograft-derived cell lines increased, which resulted in a statistically significant difference between the parental cell line NSTS-11 and the tertiary xenograft-derived cell line LTB24. This increase in CSC number was documented by the enhanced ALDH activity as well as high colony and sphere formation capacity of LTB24 cells. As these functional assays for CSC phenotype were performed using biological replicates from different passages in vitro, the obtained data also underpinned the stability of CSC numbers during short-term in vitro culture. Such stability was further demonstrated by similar expression patterns detected by immunofluorescence and qRT-PCR among different in vitro passages of each of the examined cell lines. Thus, the major advantage of our approach resides in the continuous supply of xenograft-derived cell lines that (i) may serve as a cost-effective model for the selection of CSCs for comparative studies, (ii) may be stored for future studies, and (iii) provide enough material for subsequent analyses and assays.

One of the intriguing changes detected in our model was the increase in ALDH activity despite the decrease in ALDH1 level detected over the passages in vivo. It has only recently come to light that ALDH activity but not necessarily ALDH1 expression marks CSCs [33]. Indeed, it has been demonstrated in hematopoietic cells that ALDH1A1 deficiency does not affect Aldefluor staining [34]. Similarly, although Aldefluor positivity was associated with breast CSCs, ALDH1A1 did not correlate with Aldefluor positivity and performed poorly as a predictor of breast carcinoma progression [35]. In agreement with our results, the latter study showed that increased expression of other ALDH isoforms, e.g., ALDH6A1, correlated with high ALDH activity and was associated with metastatic disease in breast carcinoma [35]. Recently, proteomic analysis of primary and metastatic prostate cancer has also demonstrated that ALDH6A1 is highly specific to progressive metastatic disease [36]. Using our model of gradual selection of rhabdomyosarcoma CSCs, we revealed an apparent upregulation of ALDH6A1 at mRNA and protein level, which was associated with Aldefluor positivity and increased stemness, while *ALDH1A1* gene expression was markedly downregulated. Importantly, the survival analysis performed using TCGA-SARC database supported the observed decrease in *ALDH1A1* expression. We show here that it is in fact the downregulation (not upregulation) of *ALDH1A1* gene expression that is associated with aggressive tumors and strongly predicts (*p* < 0.0001) poor prognosis among various sarcomas. Notably, these results contradict the previous report by Martinez-Cruzado et al., whom used artificially transformed mesenchymal stem cells as an experimental model of sarcomagenesis and proposed that ALDH1 expression is enhanced in xenograft-derived cells and associates with sarcoma CSCs [37]. In contrast, our model utilizing primary tumor-derived cells suggests ALDH6A1 as a candidate molecule that plays a role in rhabdomyosarcoma CSCs. Indeed, the expression of *ALDH6A1* has already been identified among the genes that correlate with poor outcome in rhabdomyosarcoma patients [31]. This is in agreement with our survival analysis on soft-tissue sarcomas demonstrating that upregulated expression of *ALDH6A1* but not *ALDH1A1* or *ALDH3A1* significantly correlates with poor survival when combined with other genes identified in our model, i.e., *CDH15*, *MYOD1*, *SOX4*, *ARMCX1*, and *RYK*.

Comparing the gene expression profiles of parental NSTS-11 cells and three subsequent xenograft-derived cell lines, we were able to identify gradual changes in the expression of several genes that confer stemness and/or are linked with undifferentiated myogenic precursors. For instance, our data showed an apparent increase in the expression of the *CDH15* gene, which encodes M-cadherin. In a model of RAS-driven rhabdomyosarcoma tumorigenesis, expression of *KRAS* under the *CDH15* promoter resulted in less differentiated and more aggressive tumors [38]. Cells isolated from such rhabdomyosarcoma tumor were enriched for tumor-initiating activity and expressed markers of early myoblasts and adult muscle stem cells, i.e., satellite cells [38]. Indeed, M-cadherin expression has been clearly demonstrated to be a marker of satellite cells [39], which share many similarities with the gene expression signature of embryonal rhabdomyosarcoma [40].

Although embryonal rhabdomyosarcoma most likely arises from myoblasts, both markers of quiescent satellite cells [39,41], e.g., PAX3, PAX7, or HEYL, and markers of activated satellite cells, including MYOD1 [40], are frequently expressed. Notably, *MYOD1* was one of the most upregulated genes (>8-fold in tertiary xenograft-derived LTB24 cells) in our study. MYOD1 is a transcriptional activator expressed in early muscle progenitors and is required for and regulates muscle progenitor specification [38]. In rhabdomyosarcoma, MYOD1 is expressed in small, primitive tumor cells, whereas cells that exhibit morphological evidence of skeletal muscle differentiation generally lack MYOD1 expression [42]. Recently, it has been suggested that MYOD1 regulates common gene programs that lock cells in an arrested myogenic fate and is required for the self-renewal of rhabdomyosarcoma cells and sustained tumor growth [43]. Corroborating the increase in the expression of *CDH15* along with *MYOD1* in our model of rhabdomyosarcoma CSCs, *MYOD1* knockdown has been demonstrated to significantly downregulate *CDH15* expression [43].

MYOD1 may also maintain pre-myogenic mesoderm expression by upregulating PAX3, PAX7, and EYA2 [44]. PAX3 has been shown to prevent the differentiation of myoblasts and satellite cells [22]. PAX3 also acts as an upstream regulator maintaining stemness in neural crest cells [22], which suggests its general impact on cell fate. Thus, upregulated expression of *PAX3* might explain the increased stemness of LTB24 rhabdomyosarcoma cells in our study. The gradual increase in rhabdomyosarcoma stemness could be further substantiated by the detected downregulation of 10 out of 18 genes that are associated with mesenchymal stem cell senescence [25] and by the upregulation of genes involved in early mesenchymal development and maintenance of myogenic precursors, i.e., *SOX4* [45,46] and PITX2 [23]. *SOX4* has been previously demonstrated to contribute to tumor progression by promoting cancer stemness [46]. Importantly, SOX4 is highly expressed in embryonal rhabdomyosarcoma compared with normal muscle and knockdown of SOX4 leads to a significant decrease in MYOD1 levels and impaired rhabdomyosarcoma cell survival [47]. Conversely, we have identified downregulation of *MIR29A* and *MIR145* genes encoding microRNAs that are crucial for induction of myogenic differentiation [48] and repression of pluripotency [8,25], respectively. Although downregulation of microRNA 145 (miR-145) has been suggested in tumorigenesis of Ewing’s sarcoma [8,49], our study provides the first evidence that miR-145 might be involved in regulation of CSC phenotype in rhabdomyosarcoma. Together, the gene expression profiling data indicate that serial xenotransplantation in NSG mice resulted in the progressive dedifferentiation of NSTS-11 rhabdomyosarcoma cells and their reprogramming towards the expression signature of non-senescent myogenic precursors, which was associated with enhanced rhabdomyosarcoma stemness both in vivo and in vitro.

Finally, the induction of stemness through serial xenotransplantation in NSG mice was also associated with the modulated expression of many genes involved in the EMT program. Intriguingly, functional analysis of these genes suggested an acquisition of an equilibrium between EMT and its reverse process, mesenchymal-epithelial transition (MET). Moreover, some of the typical markers of an epithelial phenotype (*CDH1*) or a mesenchymal phenotype (*CDH2*) were downregulated, which suggests a loss of differentiation and a shift towards an undifferentiated metastable phenotype that combines partial mesenchymal and epithelial traits. In carcinomas, recent accumulating evidence has shaped a widely accepted view that EMT generates CSCs, which appear to reside at an intermediate state along the epithelial-mesenchymal spectrum of phenotypes [49,50]. Carcinoma CSCs thus most likely undergo EMT only partially, attaining a hybrid epithelial/mesenchymal phenotype, which confers plasticity, invasiveness and tumor-initiating capacity [49,50]. In fact, hybrid epithelial/mesenchymal cells can integrate various epithelial and mesenchymal traits that facilitate collective cell migration and promote metastasis [51]. Corroborating these observations, excessive activation of the EMT program leading to a highly mesenchymal phenotype has been shown to be detrimental to the tumorigenic activity of CSCs [52,53]. In parallel to the results obtained in carcinomas, partial MET has been discussed as a process contributing to the metastasis and stemness of sarcomas, although this mechanism remains elusive [54,55,56].

Here, we report novel evidence for the prospective role of MET in tumors of mesenchymal origin. Notably, some of the identified rhabdomyosarcoma CSC-specific targets have already been linked with MET. For instance, it has been demonstrated that overexpression of PAX3 induces MET in mesenchymal cells [57]. Importantly, MET has also been established as a key cellular mechanism in the process of reprogramming mesenchymal somatic cells, fibroblasts, towards pluripotency induced by transcription factors, including SOX2 [58,59]. These results suggest that enhanced expression of these stemness-related factors may, in the context of embryonal rhabdomyosarcoma, result in a partial loss of mesenchymal phenotype, as indicated here by the progressive downregulation of a prominent EMT inducer, *SNAI1*, and *CDH2*, which encodes N-cadherin.

Among sarcomas, osteosarcoma tumor tissues have been shown to exhibit extremely low expression levels of N-cadherin [60]. Furthermore, several cadherins, including P-cadherin, E-cadherin, and N-cadherin, are markedly downregulated in osteosarcoma cell lines in vitro [61], which is in line with the gene expression signature associated with the enhanced rhabdomyosarcoma stemness in our study. Mechanistically, N-cadherin overexpression has been demonstrated to significantly impair osteosarcoma migration in vitro and metastasis in vivo [61]. Experimental re-expression of N-cadherin also restores cell-cell contacts and inhibits cell migration in glioma, another highly aggressive non-epithelial tumor, which is frequently characterized by low levels of N-cadherin [62]. Similarly, metastatic dissemination of neuroblastoma is strongly correlated with low N-cadherin expression [63]. Notably, N-cadherin downregulation is crucial for the migration of neural crest cells [64] and smooth muscle cells [65], which offers an explanation for how partial MET, i.e., a loss of the typical mesenchymal marker, N-cadherin, may promote the progression of sarcomas towards a more aggressive phenotype.

Although epithelial differentiation has been reported in various sarcomas, it has never been associated with a full transition of sarcoma cells to the epithelial state and rather presents a hallmark of phenotypic plasticity resulting from the active MET program [54]. In fact, the combined presence of epithelial and mesenchymal features has been proposed to contribute to aggressive sarcomas [55]. From this perspective, EMT-related signaling that would counteract complete MET is crucial for the maintenance of mesenchymal traits. Indeed, such activation of the EMT program has been identified in the present study. In addition to the MET-related gene expression signature, the increase in rhabdomyosarcoma CSC number during serial xenotransplantation correlated with the upregulation of a typical EMT-inducing gene, *ZEB1*, and a stemness-associated gene, *SOX4*, which has recently been identified as a master inducer of EMT, controlling several EMT-relevant genes [46,66]. Based on the results reported here, we hypothesize that it is the activation of both MET and EMT signaling that sets the rhabdomyosarcoma cells in a “ready-to-act” stem-like state in which they can easily exploit various microenvironmental cues to promote their survival, proliferation, and/or migration. Remarkably, another evidence supporting this hypothesis has been recently reported in a mouse model demonstrating that rhabdomyosarcoma CSCs arise from genomically instable satellite cells, which undergo MET-like process [67]. Further mechanistic studies to examine the prospective link between the hybrid epithelial/mesenchymal phenotype and rhabdomyosarcoma progression are therefore needed to advance our understanding of this rare but aggressive disease.

## 4. Materials and Methods

### 4.1. Cell Lines and Tumor Samples

The NSTS-11 cell line derived from a primary embryonal rhabdomyosarcoma was originally described in our previous studies [3,5]. Written informed consent was obtained from the patient or patient’s parents, and the primary tumor tissue was collected in accordance with the study protocol (#12/Si/2011) approved by The Research Ethics Committee of the School of Science (Masaryk University). During the serial xenotransplantation of NSTS-11 cells in vivo, 45 xenograft tumors were collected, and 45 cell lines were successfully established from these xenografts (one cell line from each xenograft tumor). Three cell lines, LTB1, LTB5, and LTB24, derived from respective xenograft tumors after each of the three subsequent passages in vivo were included for detailed in vitro analyses. All cell lines were maintained in DMEM supplemented with 20% fetal calf serum under standard conditions as described previously [3]. For the in vivo tumorigenicity assay, cell passage numbers 8–12 were used according to the growth characteristics of the respective cell line. Cell passage numbers 10–15 were used in other experiments; NSTS-11 cells were used up to passage 20 for some biological replicates.

### 4.2. In Vivo Tumorigenicity Assay

For each cell line, three 8-week-old female NSG (NOD/ShiLtSz-*scid*/*Il2rγ*^null^) mice were injected subcutaneously in the neck region with a suspension of 3 × 10^5^ enzymatically dissociated cells in 100 μL of serum-free DMEM. The mice were examined every three days for the presence of subcutaneous tumors. After the appearance of a tumor, the mice were sacrificed, and the tumors were excised. All animal experiments were conducted in accordance with a study (21379/2011-30) approved by the Institutional Animal Care and Use Committee of Masaryk University and registered by the Ministry of Agriculture of the Czech Republic as required by national legislation. The tumors were photographed, and the final tumor volume was measured using the following formula:

tumor volume (mm^3^) = length (mm) × width (mm) × width (mm) × 1/2

Each tumor was divided into two equal portions: one portion was processed for primary culture [3], and the second portion was fixed in 10% buffered formalin for 24 h, routinely processed for histological examination and embedded in paraffin. FFPE samples were stained with hematoxylin-eosin and examined using an Olympus BX51 microscope. IHC was performed as described below.

### 4.3. Colony Formation Assay

Cells cultured in vitro were harvested, enzymatically dissociated with Accutase^®^ (Gibco, Thermo Fisher Scientific, Waltham, MA, USA) to obtain single-cell suspension, and seeded at a density of 1000 cells per 10-cm culture dish (Sarstedt AG &Co, Numbrecht, Germany). Colonies were monitored to ensure they were derived from single cells. After 8 days of culture under standard conditions (see Section 4.1), cells were fixed with methanol and stained using Coomassie Brilliant Blue R-250 (Sigma-Aldrich, St. Louis, MO, USA) [68]. The whole culture dish was examined under a phase contrast Olympus CKX41 light microscope and the number of cells within individual colonies was determined by manual counting the nuclei of Coomassie-stained cells under a 20× objective. Only colonies that contained a minimum of 50 cells were considered for further analysis.

### 4.4. Sphere Formation Assay

For the sphere formation assay, cells were harvested and enzymatically dissociated with Accutase^®^ (Gibco) to obtain a single-cell suspension. Cells were resuspended in a defined serum-free medium (DMEM/F12 (GE Healthcare Europe GmbH, Freiburg, Germany) supplemented with 10 ng/mL EGF (Sigma-Aldrich), 20 ng/mL FGF2 (Sigma-Aldrich), and B-27 supplement w/o vitamin A (Gibco)) and plated in triplicates into ultra-low attachment 6-well plates (Corning, Corning, NY, USA) at a density of 2000 cells/well. Every three days, growth factors were replenished with fresh culture medium. After two weeks, rhabdospheres were counted under an Olympus CKX41 light microscope.

### 4.5. Aldefluor Assay

The Aldefluor™ assay was performed according to the kit manufacturer’s instructions (Stem Cell Technologies, Grenoble, France). Briefly, cells were suspended in Aldefluor™ assay buffer (1 × 10^4^ cells/mL) containing ALDH substrate (BODIPY-aminoacetaldehyde) and incubated at 37 °C for 45 min. Control samples were incubated in a buffer containing a specific ALDH inhibitor, diethylaminobenzaldehyde (DEAB). The fluorescence signal was measured using a BD FACS Verse flow cytometer, and ALDH activity was analyzed with BD FACSuite software (both BD Biosciences, San Diego, CA, USA).

### 4.6. Immunohistochemistry

For immunohistochemical analysis, primary tumor tissue and three sets of xenograft tumor tissues collected during subsequent xenotransplantations in NSG mice were used. Four-micron-thick tissue sections were first deparaffinized with xylene and rehydrated through a graded alcohol series. Endogenous peroxidase activity was quenched with 3% hydrogen peroxide for 10 min. For the detection of OCT4, CD133, SOX2, nestin, and NANOG, heat-induced epitope retrieval was performed in a Decloaking Chamber NxGen (Biocare Medical, Pacheco, CA, USA) at 95 °C for 40 min using citrate (pH 6.0) or Tris/EDTA (pH 9.0) buffer (Dako, Glostrup, Denmark) (Table S2). For ABCG2 staining, the sections were not pretreated. Next, the slides were incubated with the indicated primary antibody at room temperature for 60 min. Subsequently, a streptavidin-biotin horseradish peroxidase complex (Vectastain Elite ABC Kit; Vector Laboratories, Burlingame, CA, USA) or a two-step detection system without avidin and biotin (EnVision+ Dual Link system-HRP; Dako) was applied. All slides were immersed in 3,3′-diaminobenzidine (DAB; Dako) and counterstained with Gill’s hematoxylin. Negative controls were prepared by incubating samples without primary antibody. Antibody specifications, dilutions, pretreatments, detection systems and positive controls are listed in Table S2.

All immunostained slides were evaluated using a compact research microscope (Nikon ECLIPSE Ci-E). At least five discrete foci of neoplastic infiltration were selected, and the proportion of positive tumor cells was scored as follows: 0 (0%), 1 (1–5%), 2 (6–20%), 3 (21–50%), and 4 (51–100%). The immunoreactivity of tumor cells was graded as - (none), + (weak), ++ (medium), and +++ (strong).

### 4.7. Immunofluorescence

Indirect immunofluorescence (IF) was performed as previously described [3]. The primary and secondary antibodies that were used in these experiments are listed in Table S3; mouse monoclonal anti-α-tubulin served as the positive control. An Olympus BX-51 microscope was used for sample evaluation; images were captured using an Olympus DP72 CCD camera and were analyzed using the Cell^P imaging system (Olympus, Tokyo, Japan). The samples were prepared from at least three independent passages of all examined cell lines, and at least 200 cells were evaluated in each sample. The immunoreactivity and the percentage of cells showing positivity for the examined antigen were determined. Finally, for each cell line, the total immunoscores were calculated for individual antigens by multiplying the percentage of positive cells by the respective immunoreactivity as described previously [69].

### 4.8. Western Blotting

ALDH1 and ALDH6A1 protein expression was analyzed per our standard procedure [3]. The primary and secondary antibodies used are listed in Table S3; mouse monoclonal anti-β-actin and rabbit monoclonal anti-glyceraldehyde-3-phosphate dehydrogenase (GAPDH) served as loading controls. Densitometry was performed using Fiji software [70]. Band densities for protein of interest were normalized to that of the band for β-actin or GAPDH in the same sample.

### 4.9. RT-PCR and qRT-PCR

For both RT-PCR and qRT-PCR, total RNA was extracted and reverse transcribed as previously described [71]. For end point PCR, the reaction mixture (25 μL) contained 100 μM of deoxynucleoside triphosphate (dNTP) mixture, 1 U of Taq polymerase (Top-Bio, Vestec, Czech Republic), 0.25 μM of each primer and 10 μL of cDNA. The amplification was performed for 35 cycles of 94 °C for 30 s, 60 °C for 30 s, and 72 °C for 45 s.

For microarray validation experiments, qPCR was performed using the KAPA SYBR^®^ FAST qPCR Kit (Kapa Biosystems, Wilmington, MA, USA) and 7500 Fast Real-Time PCR System (Applied Biosystems, Foster City, CA, USA) as previously described [10]. The expression of individual genes was assessed using at least three technical replicates from three biological replicates (different cell passages in vitro) of each cell line. The heat shock protein gene *HSP90AB1* was used as the endogenous reference control, and the level of gene expression was normalized to that in parental NSTS-11 cells. The primer sequences used are listed in Table S4.

### 4.10. Expression Profiling

Total RNA was extracted and processed through an Affymetrix workflow as previously described [10]. Raw microarray data are available in the ArrayExpress database (www.ebi.ac.uk/arrayexpress) under accession number E-MTAB-7664. Affymetrix power tools were used to normalize raw CEL files at the gene level. Robust multiarray averaging (RMA) normalization and complete annotation files were selected.

Gene expression fold changes (FCs) were calculated for each xenograft-derived cell line using the parental NSTS-11 cell line as a reference control. Thus, gene expression FCs represent the differences in gene expression after each passage in vivo: first passage (LTB1/NSTS-11), second passage (LTB5/NSTS-11), and third passage (LTB24/NSTS-11).

To identify genes that exhibited a downward or an upward trend of expression during serial xenotransplantation, the following approach was used. First, reference fold-change values, which reflected a mean decrease or increase in gene expression after each passage in vivo, were calculated based on a set of significantly downregulated genes (*n* = 55) or a set of significantly upregulated genes (*n* = 55), respectively (Table S1). The set of significantly downregulated genes was identified using the following parameters: FC ≤ 0.25 after the third passage in vivo and FC ≤ 1.5 after any passage in vivo. Conversely, significantly upregulated genes were defined as follows: FC ≥ 4 after the third passage in vivo and FC ≥ 0.66 after any passage in vivo.

The calculated reference fold-change values were then used to determine the Pearson’s correlation coefficient of each gene. Only differentially expressed genes (≥1.5-fold after the third passage in vivo) with a strong correlation coefficient (>0.8) were included in further analyses (Table S1). Genes with FC >1.5 or FC <0.66 after the first passage in vivo were excluded from the group of downregulated or upregulated genes, respectively. The DAVID annotation tool [72] was used for gene ontology analysis, and expression heat maps were prepared using the visualization and analysis software Morpheus (https://software.broadinstitute.org/morpheus).

### 4.11. Phospho-Protein Arrays

The relative phosphorylation levels of 49 RTKs and 26 downstream kinases, including 9 MAPKs, were analyzed using the Human Phospho-RTK Array kit and the Human Phospho-MAPK Array kit (both R&D Systems, Minneapolis, MN, USA), respectively, according to the manufacturer’s protocol. Each array was incubated with 250 μg of protein lysate. The levels of phosphorylation were quantified using Fiji software [70]. Pixel densities of duplicated spots were averaged, and the value of background was subtracted. The analysis was performed as previously described [71].

### 4.12. Statistical Analysis

The colony formation assay and sphere assay were analyzed using Mann-Whitney test (one-tailed), mean tumor volume increase, Aldefluor™ assay data and Western blot densitometry data were analyzed using unpaired Welch’s *t*-test (two-tailed) in GraphPad Prism 8.0.2 software (GraphPad Software Inc., San Diego, CA, USA). *p* < 0.05 was considered statistically significant.

## 5. Conclusions

In this study, we demonstrate that serial xenotransplantation in NSG mice in conjunction with short-term culture of xenograft-derived cells is an effective tool to screen for molecular targets in rhabdomyosarcoma CSCs. This approach enabled us to identify several novel and promising rhabdomyosarcoma CSC-specific targets, e.g., *ALDH6A1*, *SOX4*, *PAX3*, *CDH15*, downregulated *MIR145*, or phosphorylated RYK, which warrant validation in subsequent mechanistic studies. Most importantly, the presented model of the progressive selection of CSCs has provided the first evidence that the recently emerged link between the hybrid epithelial/mesenchymal phenotype and cancer stemness may also account for embryonal rhabdomyosarcoma. Identification of the hybrid epithelial/mesenchymal gene expression signature associated with the rhabdomyosarcoma CSC phenotype demonstrates the ability of the reported approach to unveil complex molecular changes. Deciphering these complex molecular traits might be essential to achieving a better understanding of the mechanisms underlying the induction and maintenance of stemness in cancer.

## Figures and Tables

**Table 1:** Serial xenotransplantation of NSTS-11 cells in NSG mice. A total of 3 × 10^5^ parental NSTS-11 cells or cultured cells derived from the xenograft tumors obtained in a previous in vivo passage were subcutaneously injected into the mice.

In Vivo Passage Number	Tumorigenic Efficiency		Mean Tumor Volume Increase (mm^3^/Day ± SEM)
	Mice with Tumors/Mice Injected	Tumors Formed in Total ^1^	
First	3 of 3 (100%)	3	2.89 ± 0.74 (*n* = 3)
Second	6 of 9 (67%)	9	4.37 ± 1.83 (*n* = 9)
Third	27 of 27 (100%)	33	5.66 ± 0.72 (*n* = 27) *

^1^ In several mice, subcutaneously injected cells formed two spatially separated tumors within the site of injection and these tumors were handled separately in subsequent experiments. SEM, standard error of the mean; n, number of mice evaluated. * Statistically significant compared with the first in vivo passage (*p* < 0.05; Welch’s *t*-test).

**Table 2:** Immunohistochemical analysis of primary tumor tissue and xenograft tumor tissues.

Antigen	Primary Tumor		Primary Xenografts ^1^		Secondary Xenografts ^1^		Tertiary Xenografts ^1^	
	%TC	IR	%TC	IR	%TC	IR	%TC	IR
SOX2	2	+++	3	+++	3	++	3	++
OCT4	1	+	1	+	1	+	1	+
NANOG	0	-	0	-	0	-	0	-
Nestin	4	+++	4	+++	4	+++	4	+++
CD133	3	+	1	+	1	+	1	+
ABCG2	1	++	1	++	1	+	1	+

The percentage of positive tumor cells (%TC) was categorized into five levels: 0 (0%), 1 (1–5%), 2 (6–20%), 3 (21–50%), and 4 (51–100%). The immunoreactivity of tumor cells (IR) was graded as - (none), + (weak), ++ (medium), and +++ (strong). ^1^ Data are presented as the mean of three independent xenograft tumor tissues representing the three arms of the serial xenotransplantation of NSTS-11 cells derived from the primary tumor.

## Supplementary Materials

The following are available online at https://www.mdpi.com/2072-6694/12/1/196/s1, Figure S1: Uncropped Western blot images of ALDH1 immunodetection, Figure S2: Uncropped Western blot images of ALDH6A1 immunodetection, Figure S3: IHC analysis of the expression of SOX2, OCT4, and NANOG in primary and xenograft tumor tissues, Figure S4: IHC analysis of the expression of nestin, CD133, and ABCG2 in primary and xenograft tumor tissues, Figure S5: Immunofluorescence analysis of the expression of SOX2, OCT4, and NANOG in the primary tumor-derived NSTS-11 cell line and the primary, secondary, and tertiary xenograft tumor-derived cell lines, LTB1, LTB5, and LTB24, respectively, Figure S6: Immunofluorescence analysis of the expression of nestin, CD133, and ABCG2 in the primary tumor-derived NSTS-11 cell line and the primary, secondary, and tertiary xenograft tumor-derived cell lines, LTB1, LTB5, and LTB24, respectively, Figure S7: *SOX2*, *POU5F1* (OCT4), *NANOG*, *NES* (nestin), *PROM1* (CD133), and *ABCG2* mRNA expression levels in embryonal rhabdomyosarcoma cell lines derived from a primary tumor (NSTS-11) and subsequent xenograft tumors (LTB1, LTB5, and LTB24), Figure S8: Phosphorylation status of RTKs during serial xenotransplantation, Figure S9: Phosphorylation status of MAPK and other serine/threonine kinases, Figure S10: *SOX2* expression significantly correlates with poor survival in soft-tissue sarcoma patients, Table S1: Gene expression profiling data and analyses, Table S2: Antibodies and detection reagents used for immunohistochemistry, Table S3: Antibodies used for immunofluorescence and Western blotting, Table S4: Primers used for RT-PCR and qRT-PCR.
