# Supplementary material for: Serial Xenotransplantation in NSG Mice Promotes a Hybrid Epithelial/Mesenchymal Gene Expression Signature and Stemness in Rhabdomyosarcoma Cells
Source: Cancers (Basel). 2020 Jan 13;12(1):196. doi: 10.3390/cancers12010196 (PMC7016569; doi:10.3390/cancers12010196)
Supplement: Supplementary file 1 [file cancers-12-00196-s001.zip › Skoda_et_al_Cancers_Supplementary_Materials.pdf]

cancers

MDPI

Supplementary Materials

Serial xenotransplantation in NSG mice promotes a hybrid epithelial/mesenchymal gene expression signature and stemness in rhabdomyosarcoma cells

Jan Skoda, Jakub Neradil, Iva Staniczkova Zambo, Alena Nunukova, Peter Macsek, Karolina Borankova, Viera Dobrotkova, Pavel Nemec, Jaroslav Sterba and Renata Veselska

Contents

Supplementary Figures

Figure S1. Uncropped Western blot images of ALDH1 immunodetection.

Figure S2. Uncropped Western blot images of ALDH6A1 immunodetection.

Figure S3. IHC analysis of the expression of SOX2, OCT4, and NANOG in primary and xenograft tumor tissues.

Figure S4. IHC analysis of the expression of nestin, CD133, and ABCG2 in primary and xenograft tumor tissues.

Figure S5. Immunofluorescence analysis of the expression of SOX2, OCT4, and NANOG in the primary tumor-derived NSTS-11 cell line and the primary, secondary, and tertiary xenograft tumor-derived cell lines, LTB1, LTB5, and LTB24, respectively.

Figure S6. Immunofluorescence analysis of the expression of nestin, CD133, and ABCG2 in the primary tumor-derived NSTS-11 cell line and the primary, secondary, and tertiary xenograft tumor-derived cell lines, LTB1, LTB5, and LTB24, respectively.

Figure S7. *SOX2*, *POU5F1* (OCT4), *NANOG*, *NES* (nestin), *PROM1* (CD133), and *ABCG2* mRNA expression levels in embryonal rhabdomyosarcoma cell lines derived from a primary tumor (NSTS-11) and subsequent xenograft tumors (LTB1, LTB5, and LTB24).

Figure S8. Phosphorylation status of RTKs during serial xenotransplantation.

Figure S9. Phosphorylation status of MAPK and other serine/threonine kinases.

Figure S10. *SOX2* expression significantly correlates with a poor survival of soft-tissue sarcoma patients.

Supplementary Tables

Table S1 is available as a separate Excel spreadsheet file

Table S2. Antibodies and detection reagents used for immunohistochemistry.

Table S3. Antibodies used for immunofluorescence and western blotting.

Table S4. Primers used for RT-PCR and qRT-PCR.

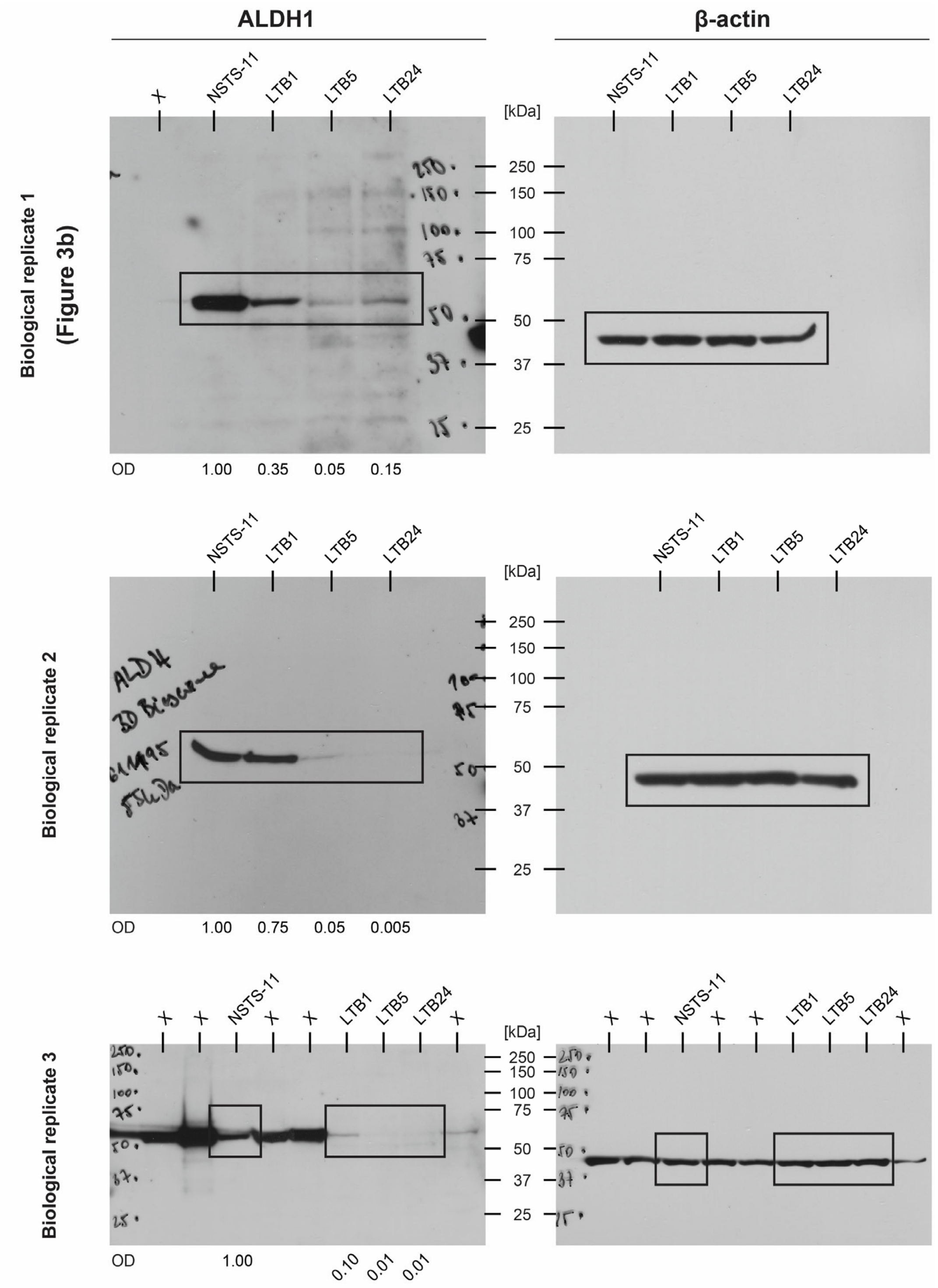

Figure S1. Uncropped Western blot images of ALDH1 immunodetection. Relative optical density (OD) values normalized to the respective β-actin loading controls are denoted for each biological replicate. In some cases, cell lysates not related to this study were probed on the same membrane – lanes marked "X".

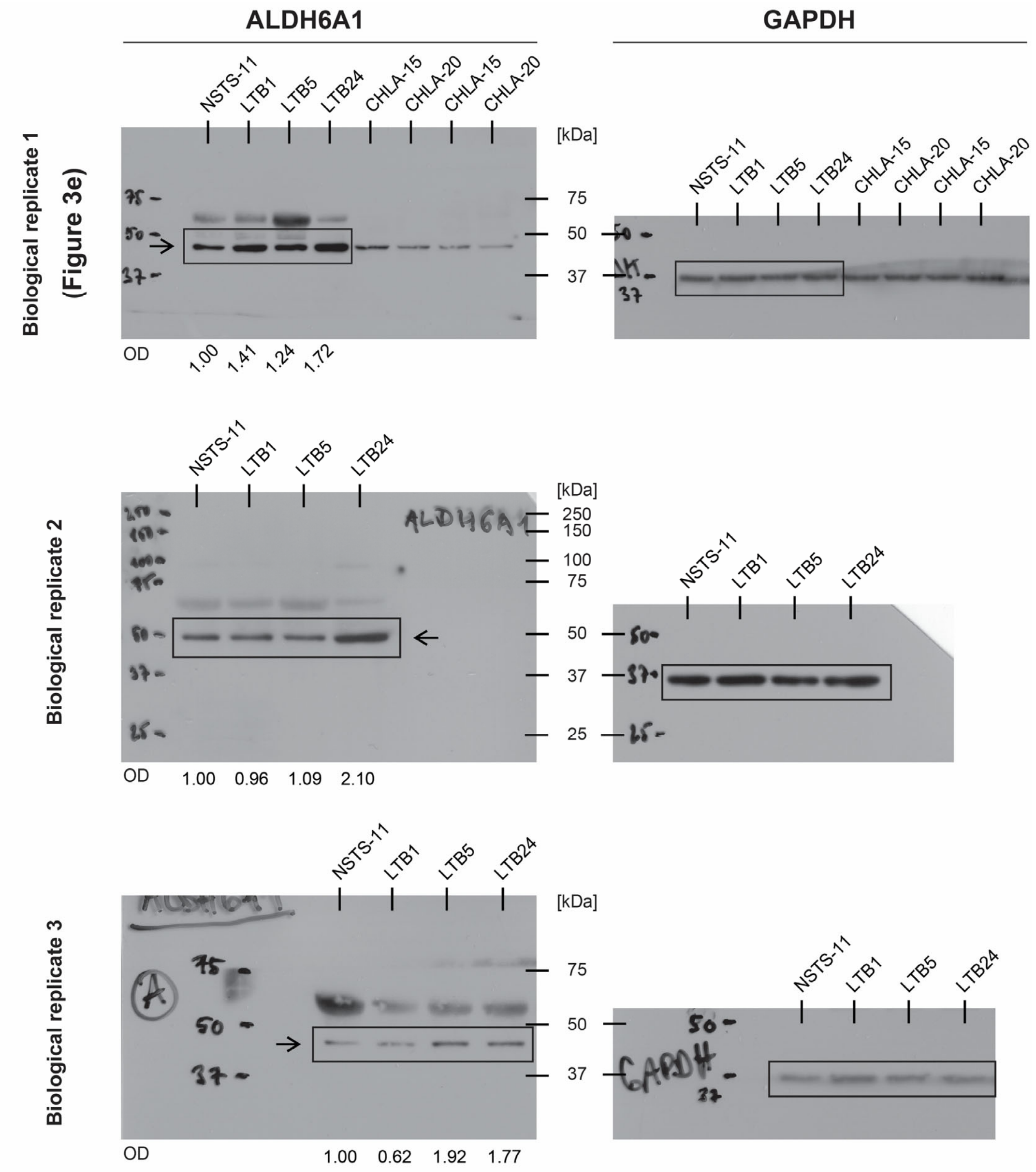

Figure S2. Uncropped Western blot images of ALDH6A1 immunodetection. Relative optical density (OD) values normalized to the respective GAPDH loading controls are denoted for each biological replicate. In the biological replicate 1, cell lysates from CHLA-15 and CHLA-20 neuroblastoma cell lines were included in the blot as independent controls to distinguish the specific ALDH6A1 band (marked by arrow).

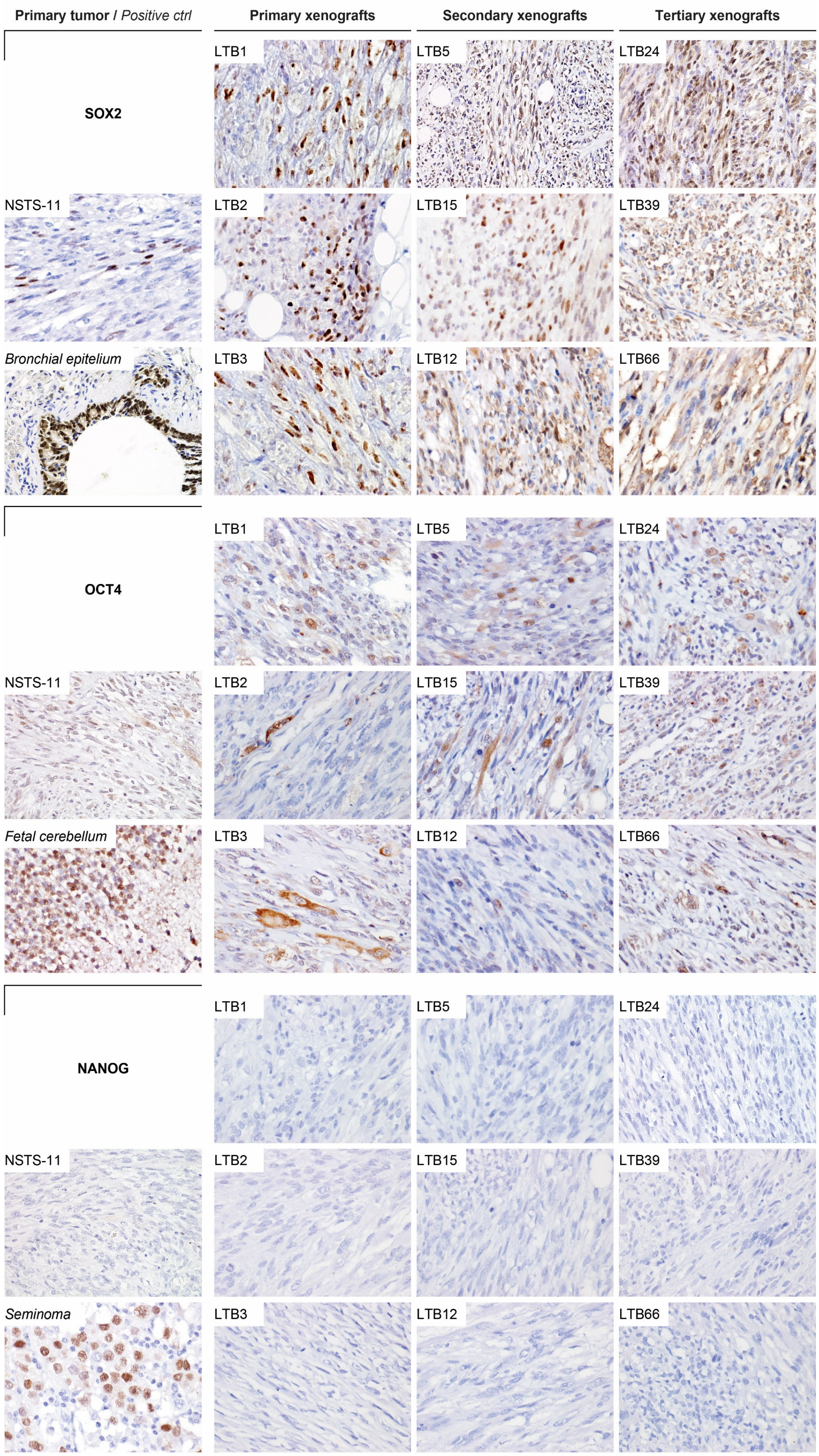

Figure S3. IHC analysis of the expression of SOX2, OCT4, and NANOG in primary and xenograft tumor tissues. Positive controls (ctrl) are indicated in *italics*. Original magnification, 400×.

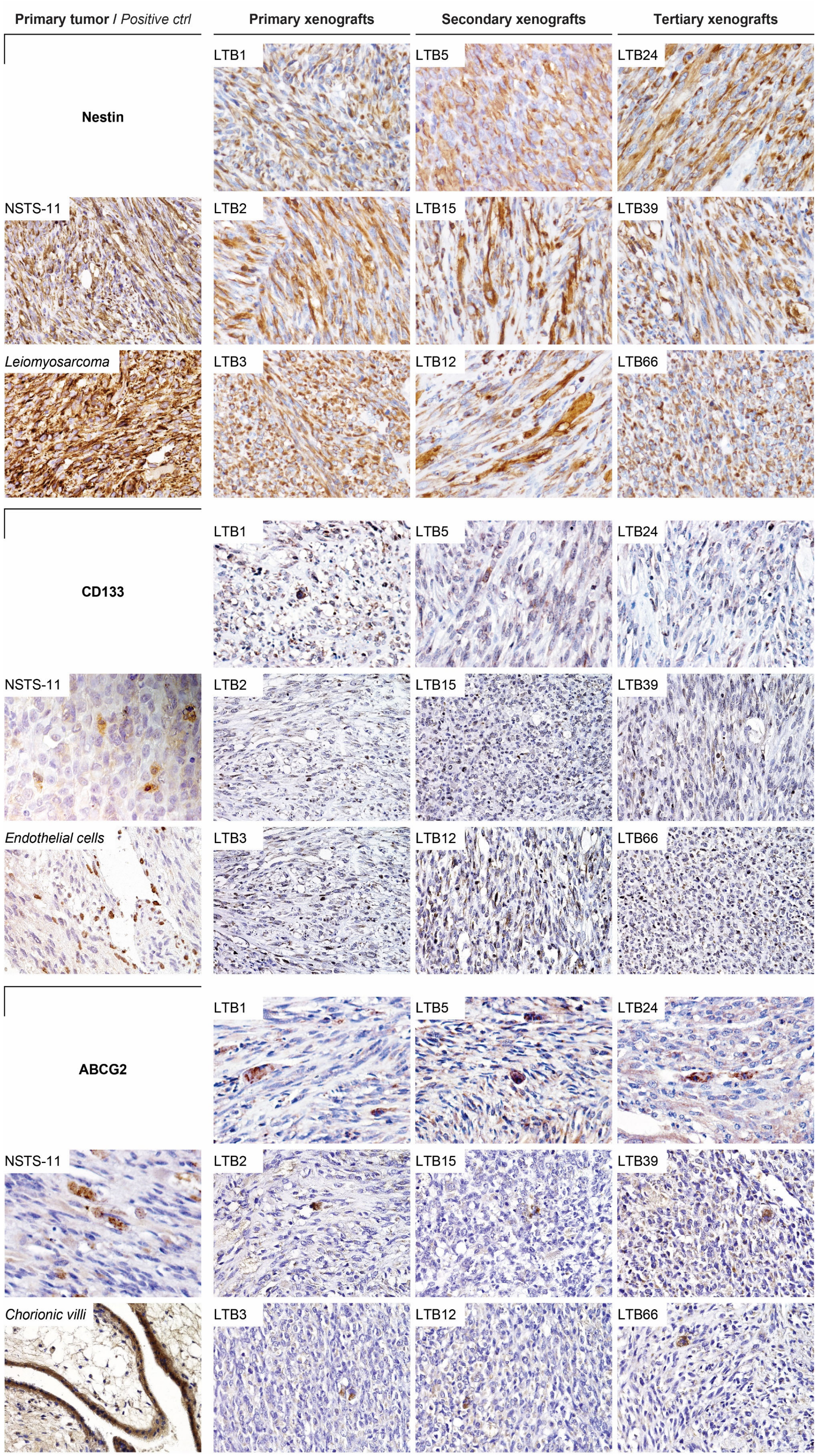

Figure S4. IHC analysis of the expression of nestin, CD133, and ABCG2 in primary and xenograft tumor tissues. Positive controls (ctrl) are indicated in *italics*. Original magnification, 400×.

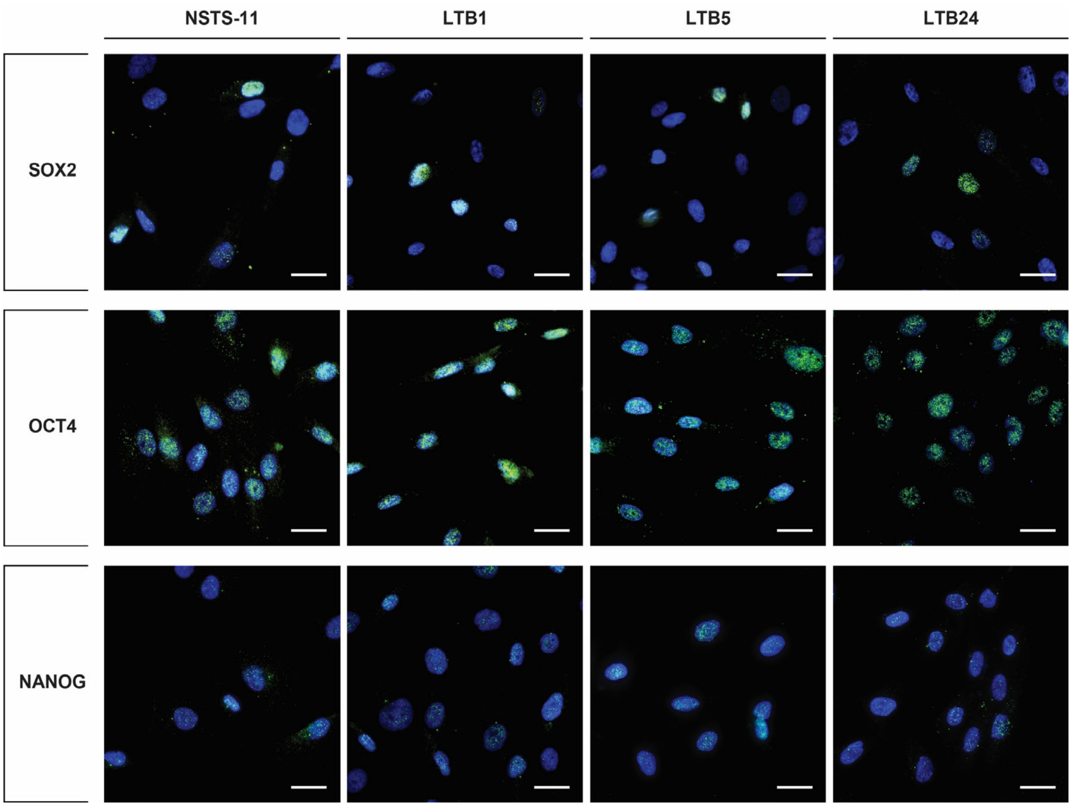

Figure S5. Immunofluorescence analysis of the expression of SOX2, OCT4, and NANOG in the primary tumor-derived NSTS-11 cell line and the primary, secondary, and tertiary xenograft tumor-derived cell lines, LTB1, LTB5, and LTB24, respectively. Each marker was visualized by indirect immunofluorescence using Alexa Fluor® 488-conjugated secondary antibody (*green*); nuclei were counterstained with DAPI (*blue*). Scale bars, 25 μm.

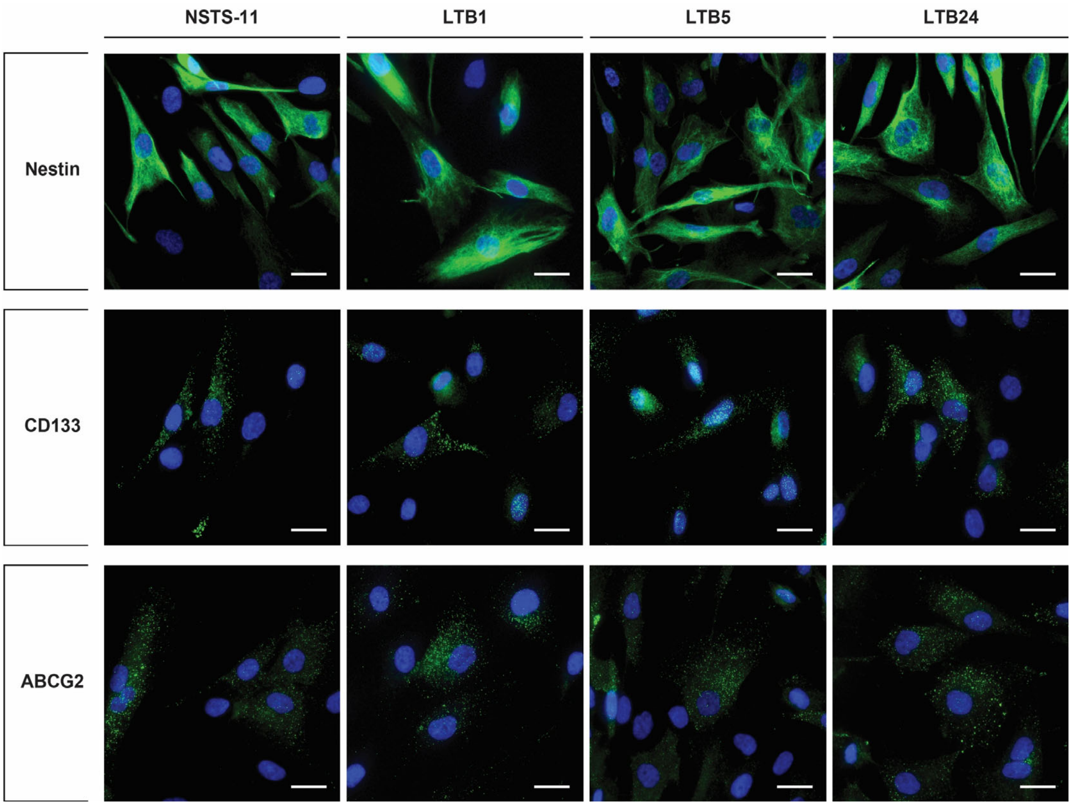

Figure S6. Immunofluorescence analysis of the expression of nestin, CD133, and ABCG2 in the primary tumor-derived NSTS-11 cell line and the primary, secondary, and tertiary xenograft tumor-derived cell lines, LTB1, LTB5, and LTB24, respectively. Each marker was visualized by indirect immunofluorescence using Alexa Fluor® 488-conjugated secondary antibody (*green*); nuclei were counterstained with DAPI (*blue*). Scale bars, 25 μm.

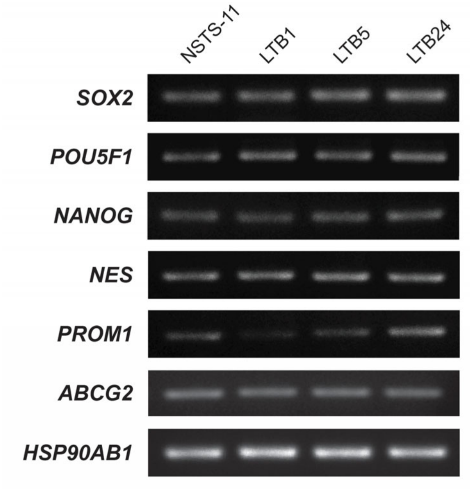

Figure S7. *SOX2*, *POU5F1* (OCT4), *NANOG*, *NES* (nestin), *PROM1* (CD133), and *ABCG2* mRNA expression levels in embryonal rhabdomyosarcoma cell lines derived from a primary tumor (NSTS-11) and subsequent xenograft tumors (LTB1, LTB5, and LTB24). HSP90AB1 was used as a control for RT-PCR.

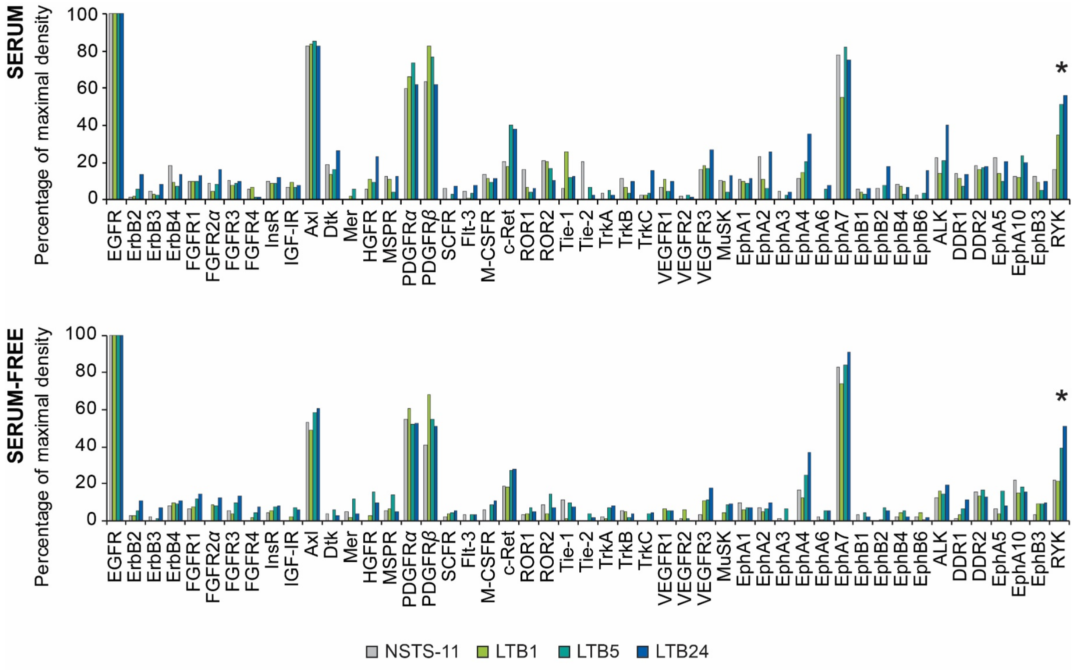

Figure S8. Phosphorylation status of RTKs during serial xenotransplantation. Regardless of the culture conditions, phosphorylation of RYK (indicated by asterisk) was consistently and significantly upregulated in cells derived from the latter xenografts LTB5 and LTB24.

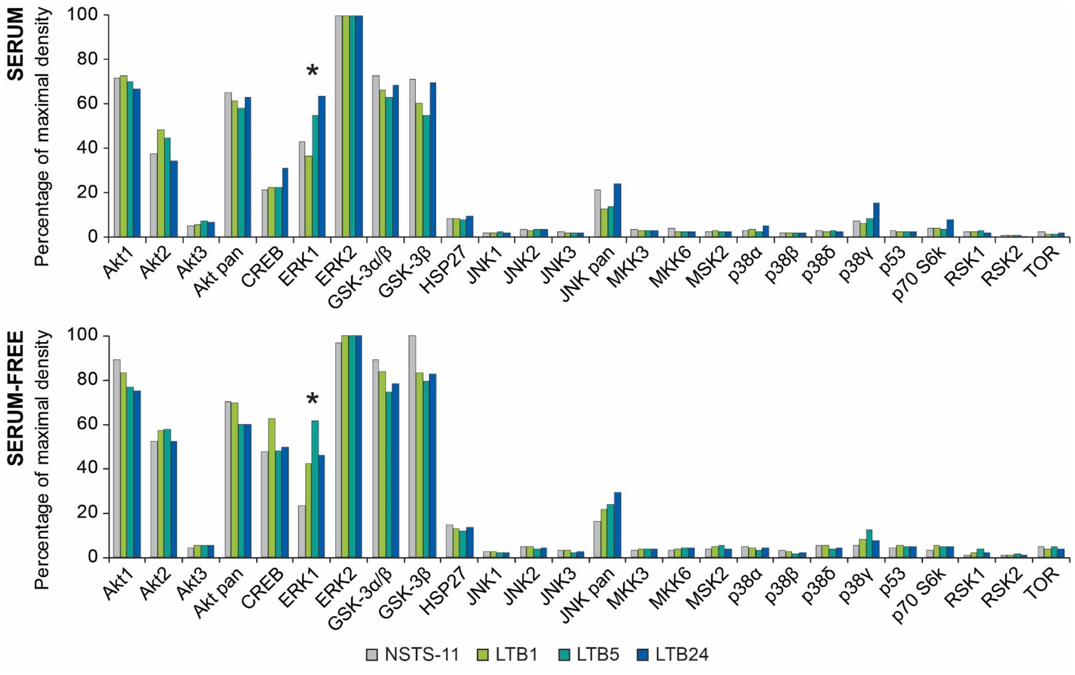

Figure S9. Phosphorylation status of MAPK and other serine/threonine kinases. Note the upregulation of phosphorylated ERK1 (indicated by asterisks) in both serum and serum-free conditions.

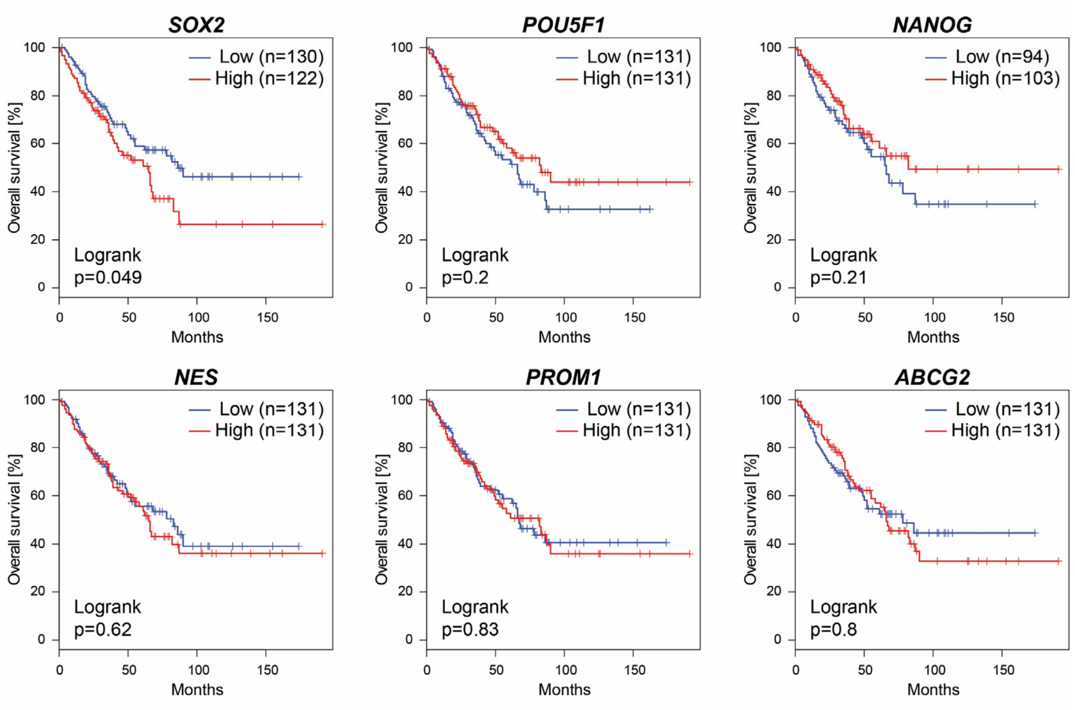

Figure S10. *SOX2* expression significantly correlates with a poor survival of soft-tissue sarcoma patients. Survival analysis based on the expression of *SOX2*, *POU5F1*, *NANOG*, *NES*, *PROM1* and *ABCG2* expression among soft-tissue sarcomas (TCGA-SARC dataset). The analysis was performed using GEPIA online tool [32].

Table S2. Antibodies and detection reagents used for immunohistochemistry.

Antibody, host & type [clone]	Manufacturer (Catalogue #)	Pretreatment buffer	Dilution	Detection/ chromogene	Positive control
SOX2, Rb mAb [EPR3131]	Abcam (ab92494)	pH 9, 95°C, 40 min	1:100	EnVision/DAB	Fetal lung
OCT4, Rb pAb	Abcam (ab19857)	pH 6, 95°C, 40 min	1:200	EnVision/DAB	Fetal cerebellum
NANOG, Rb mAb [EPR2027(2)]	Abcam (ab109250)	pH 9, 95°C, 40 min	1:200	EnVision/DAB	Seminoma
Nestin, Mo mAb [10C2]	Millipore (MAB5326)	pH 9, 95°C, 40 min	1:200	Vectastain Elite ABC HRP Kit/DAB	Leiomyo-sarcoma
CD133, Mo mAb [13A4]	Millipore (MAB4310)	pH 9, 95°C, 40 min	1:100	EnVision/DAB	Endothelial cells
ABCG2, Rb pAb, HRP conjugated	Bioss (bs-0662R-HRP)	No pretreatment	1:400	Vectastain Elite ABC kit/DAB	Chorionic villi

Rb, rabbit; Mo, mouse; mAb, monoclonal antibody; pAb, polyclonal antibody

Table S3. Antibodies used for immunofluorescence and western blotting.

Primary antibodies				
Antibody	Host/clonality [clone]	Manufacturer (Catalogue #)	Antibody dilution	
			IF	WB
SOX2	Rb/mAb [EPR3131]	Abcam (ab92494)	1:10	-
OCT4	Rb/pAb	Abcam (ab19857)	1:100	-
NANOG	Rb/mAb [EPR2027(2)]	Abcam (ab109250)	1:20	-
Nestin	Mo/mAb [10C2]	Millipore (MAB5326)	1:100	-
CD133	Mo/mAb [13A4]	Millipore (MAB4310)	1:100	-
ABCG2	Mo/mAb [5D3/CD338]	BD Biosciences (552823)	1:50	-
ALDH1	Mo/mAb [44/ALDH]	BD Biosciences (611195)	-	1:1,000
ALDH6A1	Mo/mAb [C-9]	SCBT (sc-271582)	-	1:1,000
β-actin	Mo/mAb [AC-15]	Sigma-Aldrich (A1978)	-	1:20,000
GAPDH	Rb/mAb [14C10]	Cell Signaling (2118L)	-	1:10,000
Secondary antibodies				
Host/specificity	Conjugate	Manufacturer (Catalogue #)	Antibody dilution	
			IF	WB
Goat/anti-Rb IgG	Alexa Fluor® 488	Invitrogen (A-11008)	1:200	-
Goat/anti-Mo IgG	Alexa Fluor® 488	Invitrogen (A-11008)	1:200	-
Horse/anti-Mo IgG	HRP	Cell Signaling (7076)	-	1:5,000
Goat/anti-Rb IgG	HRP	Cell Signaling (7074)	-	1:5,000

Rb, rabbit; Mo, mouse; mAb, monoclonal antibody; pAb, polyclonal antibody; HRP, horseradish peroxidase

Table S4. The sequences of primers used for RT-PCR and qRT-PCR.

Gene	Primer sequence	Product size (bp)
ABCG2	F: 5′–TCACTACTTCCTTCCTTACCCCT–3′<br>R: 5′–ACAGAAACACAACACTTGGCTG–3′	107
ALDH1A1	F: 5′–GTCAAAGGCTTCCTGCCCTA–3′<br>R: 5′–GGTTCTGATAGAGCACTTGGCT–3′	155
ANKRD1	F: 5′–GCGGAGCATCTTATCGCCT–3′<br>R: 5′–GATCCATCGGCGTCTTCCC–3′	169
CDH1	F: 5′– GCCATTCTGGGGATTCTTGGA–3′<br>R: 5′– GCTCTTTGACCACCGCTCT–3′	94
CDH2	F: 5′– ATCCAGACCGACCCAAACAG–3′<br>R: 5′– GCAGCAACAGTAAGGACAAAC–3′	95
CDH15	F: 5′– CCAACGAGGGTGTTCTGTCC–3′<br>R: 5′– TCTGCACCGACACTTTGAGTT–3′	81
HSP90AB1	F: 5′–CGCATGAAGGAGACACAGAA–3′<br>R: 5′–TCCCATCAAATTCCTTGAGC–3′	169
KRT5	F: 5′– GAGATCGCCACTTACCGCA–3′<br>R: 5′– TGCCATATCCAGAGGAAACACT–3′	115
KRT14	F: 5′– CGGCCTGCTGAGATCAAAGA–3′<br>R: 5′– ATTGTCCACTGTGGCTGTGA–3′	87
MIR145	F: 5′– CTTGTCCTCACGGTCCAGTT–3′<br>R: 5′–CCATGACCTCAAGAACAGTATTTCC–3′	83
MYOD1	F: 5′– GGGGCTAGGTTCAGCTTTCT–3′<br>R: 5′– GCTCTGGCAAAGCAACTCTT–3′	233
NANOG	F: 5′–TGAACCTCAGCTACAAACAG–3′<br>R: 5′–CTGGATGTTCTGGGTCTGGT–3′	248
NES	F: 5′–AGTGATGCCCCTTCACCTTG–3′<br>R: 5′–GCTCGCTCTCTACTTTCCCC–3′	190
PAX3	F: 5′– ACAACGCCTGACGTGGAGAA–3′<br>R: 5′– GATGCGGCTGATGGAACTCA–3′	144
POU5F1	F: 5′–TGGAGAAGGAGAAGCTGGAGCAAAA–3′<br>R: 5′–GGCAGATGGTCGTTTGGCTGAATA–3′	186
PROM1	F: 5′–CCATTGACTTCTTGGTGCTGT–3′<br>R: 5′–TGGAGTTACGCAGGTTTCTCT–3′	172
SOX2	F: 5′–AAGAGAACACCAATCCCATCC–3′<br>R: 5′–TCCAGATCTATACAAGGTCCATTC–3′	162
SOX4	F: 5′– GAAGGGAGGGGGAAACATACA–3′<br>R: 5′–CGGAATCGGCACTAAGGAGTT–3′	109
ZEB1	F: 5′– AAGAAATCCTGGGGCCTGAAG–3′<br>R: 5′– TGACCACTGGCTTCTGGTGT–3′	199

F, forward primer; R, reverse primer
